# Frond architecture of the rootless duckweed *Wolffia globosa*

**DOI:** 10.1186/s12870-021-03165-5

**Published:** 2021-08-20

**Authors:** Jingjing Yang, Xuyao Zhao, Gaojie Li, Shiqi Hu, Hongwei Hou

**Affiliations:** 1 The State Key Laboratory of Freshwater Ecology and Biotechnology, The Key Laboratory of Aquatic Biodiversity and Conservation of Chinese Academy of Sciences, Institute of Hydrobiology, Chinese Academy of Sciences, Wuhan, 430072 China; 2 Zhejiang Marine Development Research Institute, Zhoushan, 316021 China

**Keywords:** *Wolffia globosa*, Morphology, Three-dimensional structure, Light microscopy, Ultrastructure

## Abstract

**Background:**

The plant body in duckweed species has undergone reduction and simplification from the ancient *Spirodela* species towards more derived *Wolffia* species. Among the five duckweed genera, *Wolffia* members are rootless and represent the smallest and most reduced species. A better understanding of *Wolffia* frond architecture is necessary to fully explore duckweed evolution.

**Results:**

We conducted a comprehensive study of the morphology and anatomy of *Wolffia globosa*, the only *Wolffia* species in China. We first used X-ray microtomography imaging to reveal the three-dimensional and internal structure of the *W. globosa* frond. This showed that new fronds rapidly budded from the hollow reproductive pocket of the mother fronds and that several generations at various developmental stages could coexist in a single *W. globosa* frond. Using light microscopy, we observed that the meristem area of the *W. globosa* frond was located at the base of the reproductive pocket and composed of undifferentiated cells that continued to produce new buds. A single epidermal layer surrounded the *W. globosa* frond, and the mesophyll cells varied from small and dense palisade-like parenchyma cells to large, vacuolated cells from the ventral to the dorsal part. Furthermore, *W. globosa* fronds contained all the same organelles as other angiosperms; the most prominent organelles were chloroplasts with abundant starch grains.

**Conclusions:**

Our study revealed that the reproductive strategy of *W. globosa* plants enables the rapid accumulation of biomass and the wide distribution of this species in various habitats. The reduced body plan and size of *Wolffia* are consistent with our observation that relatively few cell types are present in these plants. We also propose that *W. globosa* plants are not only suitable for the study of structural reduction in higher plants, but also an ideal system to explore fundamental developmental processes of higher plants that cannot be addressed using other model plants.

**Supplementary Information:**

The online version contains supplementary material available at 10.1186/s12870-021-03165-5.

## Background

Duckweeds, aquatic monocotyledonous plants of the family *Lemnaceae*, include five genera (*Spirodela*, *Landoltia*, *Lemna*, *Wolffiella*, and *Wolffia*) with variable morphologies and living habits, propagating mostly by vegetative reproduction [1, 2]. Duckweeds have attracted attentions for their economic value and potential to ameliorate resource limitations and environmental problems [3]. For example, duckweeds are widely used for standardized toxicity testing of various water contaminants including nitrogen, phosphorus, metals, and numerous organic compounds [4, 5]. Duckweeds also possess good qualitative and quantitative nutritional profiles without detectable anti-proliferative or cytotoxic effects and could serve as a new source of human food [6]. Duckweed-based expression systems with strictly controlled formats have been developed to produce various recombinant proteins with relatively high yield [7, 8]. Duckweeds also may be valuable feedstock for biofuel production due to their high biomass and starch accumulation [9, 10]. Furthermore, their rapid growth rate, ease of cultivation and transformation, direct contact with water, and ability to adapt to environmental changes make duckweeds suitable plant models and excellent materials for physiological studies [3].

Duckweeds have undergone reduction and simplification of the plant body from the ancient *Spirodela* species towards the more-derived *Wolffia* species [11]. Among the five duckweed genera, *Wolffia* members are rootless and represent the smallest (0.5–1 mm) and most-reduced species; other duckweed species (including *Spirodela*, *Landoltia*, and *Lemna*) produce adventitious roots. DNA content estimates also vary nearly thirteen-fold among duckweed species, ranging from 158 Mbp in *Spirodela polyrhiza* to 1881Mbp in *Wolffia arrhiza* [12], and negatively correlate with body size [12–14]. The striking variation in body plan and size among duckweeds is one of the most extreme examples of structural reduction in any family. However, we lack knowledge about the mechanisms driving its occurrence within the plant kingdom.

Two studies by Landolt [11] described the morphology of the *Wolffia* genus and characterized its members based on their frond shape, frond width, number of stomata, stigma with or without pigment cells, etc. Anderson et al. [15] first reported the light and electron microscopic structure of the *W. arrhiza* frond, revealing that *W. arrhiza* fronds varied considerably from mature chlorenchymous tissue to the meristematic area where numerous daughter fronds develop. The frond structure of *Wolffia australiana* was similar to that of *W. arrhiza*; however, the *W*. *australiana* chloroplasts were concentrated in dorsal mesophyll cells [16]. White and Wise [17] revealed the differences in mesophyll anatomies between *Wolffia columbiana* and *Wolffia borealis*: the chloroplasts were located mainly in the epidermis of *W. columbiana* with a single mesophyll cell size and type, but were concentrated in the dorsal part of *W. borealis* with a steep gradient in cell size, as in *W. australiana* and *W. arrhiza*. Lemon and Posluszny [18] were the first to compare the developmental morphology of shoots in *S. polyrhiza*, *Lemna minor*, and *W. borealis*, which revealed the successive formation of new generations in these three duckweed species and their progressive simplification from *Spirodela* to *Lemna* to *Wolffia*. Sree et al. [19] reported the unique morphology of vegetative and generative propagation in *Wolffia microscopica* using light and electron microscopy. They observed flowering in different generations of *W. microscopica* at the same time [19]. Furthermore, *W. microscopica* fronds often possessed a ventral projection and a special ‘pseudoroot’ structure, in contrast to other duckweed species, which lack pseudoroots [19]. These previous studies demonstrated that members of the *Wolffia* genus vary distinctively in morphology, anatomy, growth etc. in adaption to different living environments.

*Wolffia globosa* is the only *Wolffia* species in China [20] and it has a genome size of 1300 Mbp [12]. Stable and transient transformation methods for *W. globosa* have been established [21]. Based on this efficient genetic transformation system, *W. globosa* has been used to express a protective edible vaccine antigen against fish vibriosis with high survival of vaccinated fish (63.3%), which indicated that *W. globosa* could serve as a bioreactor to produce edible vaccines [7]. *W. globosa* is also a good indicator of metal pollution in aquatic environments [22, 23]. Early studies by Landolt [11] reported the morphology of *W. globosa*. Huang [20] studied the phylogeny and genetic diversity of *W. globosa* in China based on sequencing of the *mat-K* gene and random amplified polymorphic DNA (RAPD) markers. Huang [20] also preliminaily compared the anatomy of the turion, frond, and flowering structures among *W. globosa* individuals from the field. The morphology and anatomy of *W. globosa* still need further investigation to reveal its unique characteristics that may be of importance in basic and applied research. Here, we report a comprehensive study of the morphology and anatomy of *W. globosa*. The findings provide a foundation for future research on duckweed growth, development, physiology, and evolution. Biological research on duckweeds is growing as genomes of some duckweed species have been sequenced [24]. We hope to attract more investigators and investors to join our efforts and realize the great potential of duckweed as a model system for basic and applied research in plants.

## Results

### Morphology of the *W. globosa* frond

**Fig. 1:**
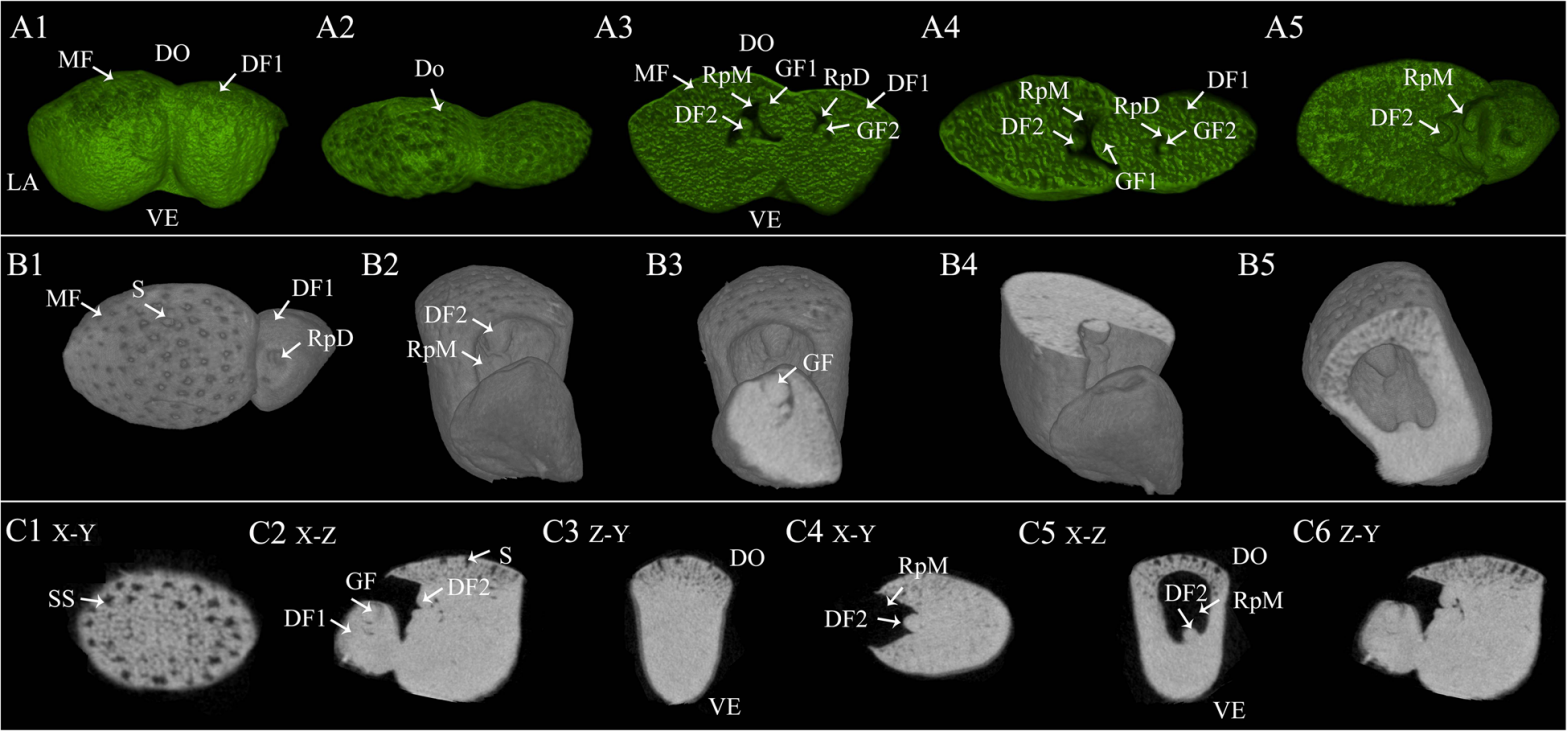
The three-dimensional volumes of *W. globosa* fronds showing the mother frond with daughter fronds. DO- dorsal part, VE- ventral part, LA- lateral part, MF- mother frond, RpM- reproductive pocket of MF, RpD- reproductive pocket of the daughter frond, DF1- the first daughter frond of MF, DF2- the second daughter frond of MF, GF1- the first daughter frond of DF1, GF2- the second daughter frond of DF1-, S- stoma, SS- substomatal cavity. Bar = 50 μm

The three-dimensional (3D) volumes of the *W. globosa* frond are shown in Fig. 1 and Movie S1. The oval-shaped *W. globosa* frond could be divided into dorsal, ventral, and lateral parts (Fig. 1A1). There was one big cavity in both the mother frond (MF) and daughter frond (DF1) named the reproductive pocket (RpM and RpD, respectively) (Fig. 1A2–A5). The MF had two visible daughter fronds (DF1 and DF2), one (DF2) budding from the base of the RpM. The DF1 also had two new buds (GF1 and GF2) (Fig. 1A3–A5). The empty RpM with the new bud (DF2) was exposed when the attached daughter frond (DF1) was separated (Fig. 1B2–B5). It was located at one end of the MF and opened when DF1 protruded from the MF. Stomata were found only in the dorsal part of the frond; no stoma were found in the ventral and lateral parts (Fig. 1 A1–A2, B1–B2). We further observed the structure of the X–Y, X–Z, and Z–Y axes at two points on the dorsal part (Fig. 1 C1–C5). Stomata and substomatal cavities were clearly observed on the dorsal side (Fig. 1 C1–C3). The RpM was one empty pouch where new generations budded (Fig. 1 C4–C5).

**Fig. 2:**
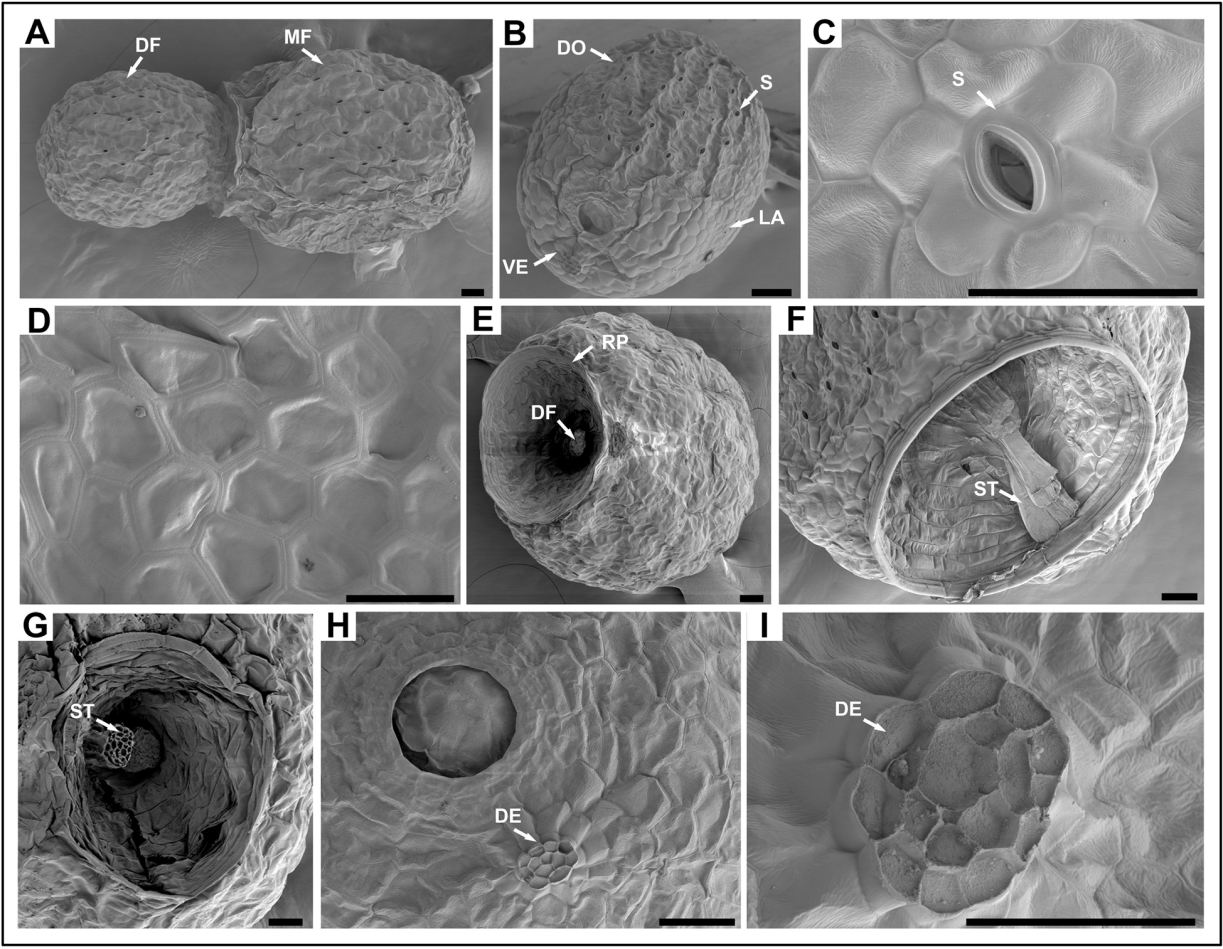
Scanning electron micrographs of *W. globosa* fronds. **A**, A single frond composed of mother frond (MF) and daughter frond (DF). **B**, The frond was divided into dorsal (DO), ventral (VE) and lateral (LA) parts. **C**–**D**, Stomata (S) and epidermal cells on the dorsal part. **E**-**G**, The daughter frond produced from reproductive pocket (RP) and connected with the mother frond by the stalk structure (ST). **H**–**I**, The detachment (DE) of the ST and its magnification. Bars = 50 μm

We also observed the morphology of the *W. globosa* frond by scanning electron microscopy (SEM). We found that the stomata were densely distributed on the dorsal surface with densities of 314.34 ± 46.99 /mm^2^ (Fig. 2 B). The guard cells, accessory guard cells, and epidermal cells made the entire stoma form an unusual flower-ring structure while the cells on the ventral and lateral parts were pentagonal (Fig. 2 C–D). The DF was released from the RpM and connected with its MF by a stalk structure (Fig. 2 E–F). The broken stalk connecting the MF and DF remained in the RpM when the DF was released. The structure of the stalk was similar to the vascular tissue of plants and filled with cavities. The other end of the stalk structure was located near the RpD and the detachment left a visible scar when the DF was released. The scar was similar in structure to the abscission layer (Fig. 2 H–I).

### Light microscopy observation

**Fig. 3:**
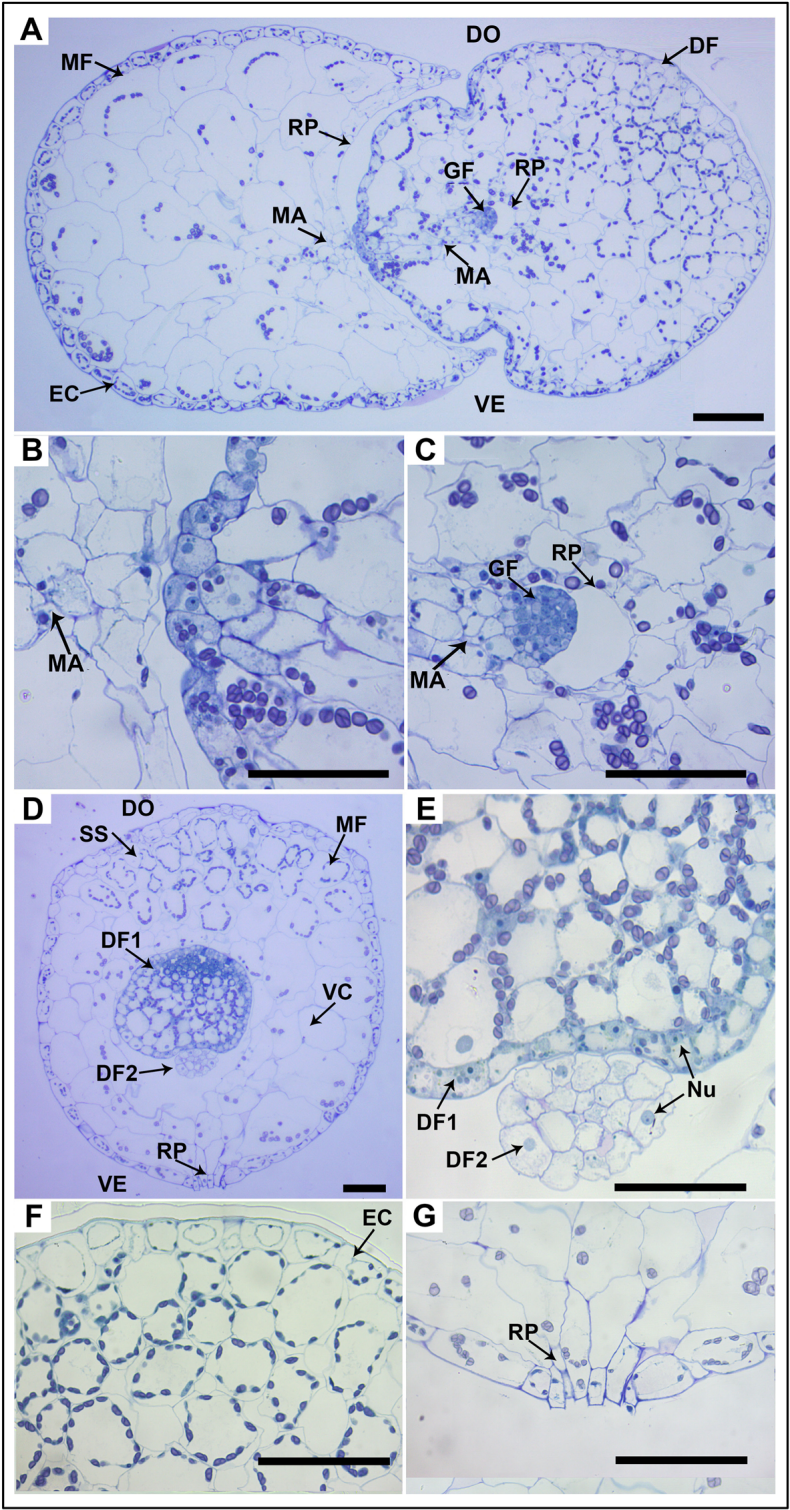
Microscopic observations of *W. globosa* fronds. **A-C**, Light micrograph of vertical cross-sections of a *W. globosa* frond showing the daughter fronds (DF, GF) were budding from the meristematic area (MA) in reproductive pocket (RP) of the mother frond (MF). DO- dorsal part, VE- ventral part. **D**, Light micrograph of horizontal cross-section of a *W. globosa* frond showing MF and daughter fronds (DF1, DF2), vacuolated cells (VC) and substomatal cavity (SS). **E**-**G**, Magnification of DF1 and DF2 with larger nuclei (NU), epidermal cells (EC) and RP. Bars = 200 μm

Horizontal and vertical cross-sections of *W. globosa* are shown in Fig. 3. We confirmed previous results that the developing DFs were produced from the meristematic area in the base of the RpM (Fig. 3 A–C). This meristematic area was composed of some undifferentiated cells that continued to multiply, producing new DFs. The RpM became larger with the growth of DFs and opened when they were released (Fig. 3 G). Most chloroplasts were concentrated in the dorsal part (Fig. 3 D). The stomata were only found in the upper epidermis (dorsal side), and prominent substomatal cavities could be observed distinctly from the vertical cross-section. From the dorsal to ventral side, the mesophyll cells varied in size and changed from small and dense palisade-like parenchyma cells to large and empty vacuolated cells with many intercellular air spaces. Furthermore, the chloroplasts showed a developmental gradient from the youngest to the oldest fronds. Compared with the MF, DFs were at an earlier differentiation stage and mainly consisted of many dividing cells with larger nuclei (Fig. 3 E). The outermost layer of the MF was composed of a single layer of epidermal cells containing annular distributed chloroplasts (Fig. 3 F).

### Ultrastructure of the *W. globosa* frond

**Fig. 4:**
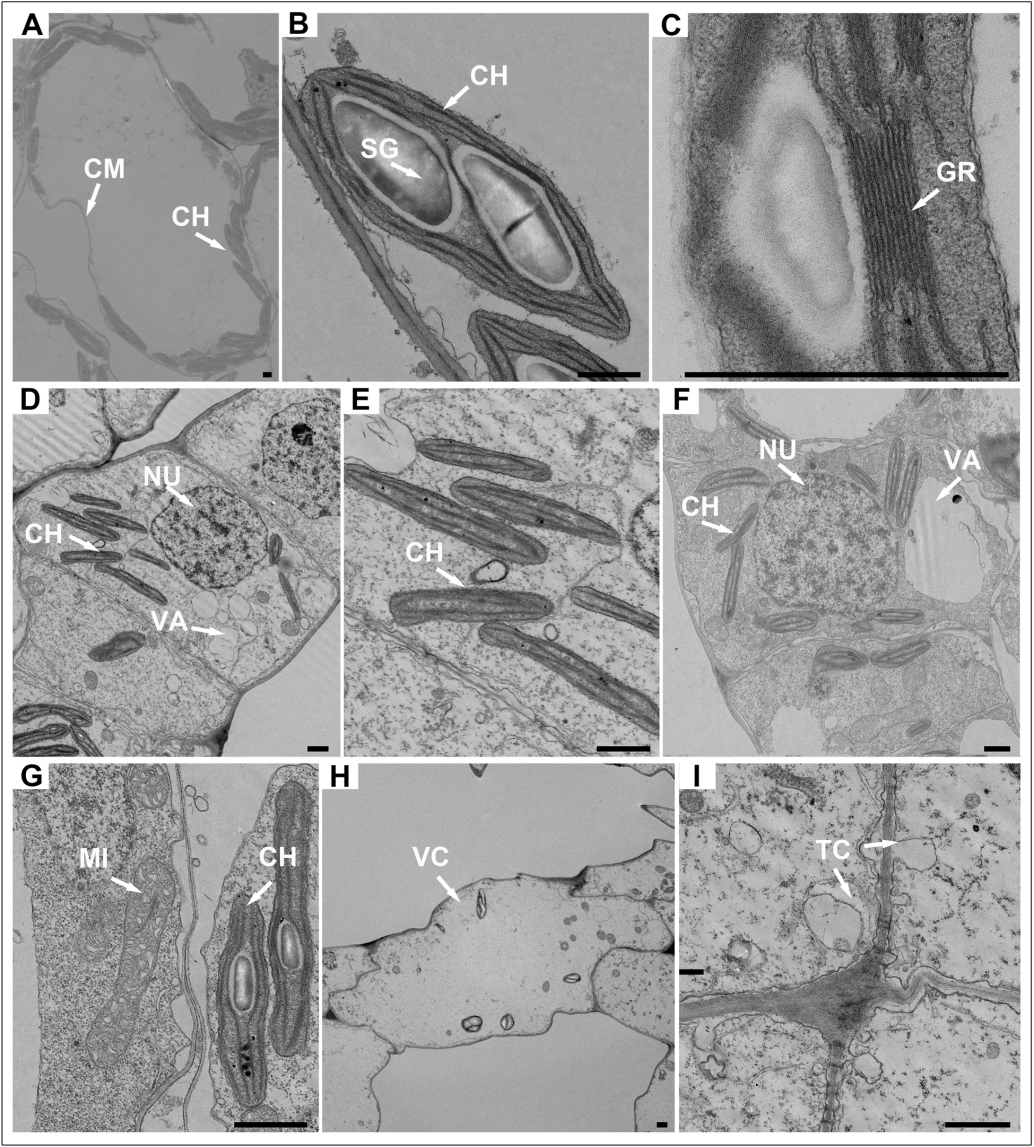
Transmission electron micrographs of *W. globosa* fronds. **A**-**B**, Lens-shaped chloroplasts (CH) containing starch grains (SG) distributed in mature mesophyll cells and their magnification, CM- cell membrane. **C**, The photosynthetic membrane system of CH containing a granum (GR) composed of thylakoids. **D**, Cell of meristematic area showing larger nuclei (NU) and smaller vacuoles (VA). **E**, The CH in meristematic area. **F**–**G**, Cell of daughter frond showing more mitochondria (MI). **H**, Cell of reproductive pocket was highly vacuolated (VC, vacuolated cells). **I**, Transfer cells (TC) in most adjacent mature mesophyll cell. Bars = 1 μm

The *W. globosa* frond contains the same organelles as other angiosperm plants (Fig. 4). The most prominent organelles were chloroplasts, which were mainly distributed in the mesophyll cells of the upper epidermis (Fig. 4 A). There were no significant differences in the size and elaboration of the thylakoid system among chloroplasts. The photosynthetic membrane system of these lens-shaped chloroplasts was well developed, and the individual grana were composed of three to eight thylakoids (Fig. 4 B–C). Starch grains occurred in the chloroplasts of almost all the palisade-like parenchyma cells but were more abundant in the chloroplasts of mature mesophyll cells than in the meristematic area or the developing DFs (Fig. 4 D–E). There were also more mitochondria in the meristematic area of the MF than in that of the DFs, which had larger nuclei and smaller vacuoles (Fig. 4 F–G). Microbodies were often, but not always, found in close association with the chloroplasts. Other organelles such as Golgi, free ribosomes, and rough endoplasmic reticulum were not so prominent. The outermost cells of the RpM were mostly vacuolated and organelles were almost invisible (Fig. 4 H). Furthermore, we found elaborate cell wall projections, which were classified as transfer cells, in most adjacent mature mesophyll cells. These transfer cells were ingrowths, increasing the area of the cell membrane (Fig. 4 I).

## Discussion

To complete their life, most plant species require all the vegetative (shoot, stem, and root) and reproductive (flower, fruit, and seed) organs. These plants produce numerous branches through the growth of the shoot apical meristem (SAM) and root apical meristem (RAM) [25]. However, the morphology of *Wolffia* fronds does not fit traditional botanical descriptions. The *W. globosa* frond normally budded new fronds from its unique meristematic area by vegetative propagation. The meristematic area of the *W. globosa* frond was located at the base of the RpM and was a collection of undifferentiated cells with the ability to divide; there were no morphologically strict divisions in the meristem area. Our finding of frond propagation in *W. globosa* is consistent with previous studies on *W. arrhiza* [15], *W. australiana* [16], *W. microscopica* [19], *W. columbiana,* and *W. borealis* [17, 18]. We speculated that the dividing cells may perform different functions than the SAM in *A. thaliana* including expression of some key genes involved in SAM activity and the distribution of auxin and cytokinin. This latter possibility could be investigated using reporter genes based on the established genetic transformation system for *W. arrhiza* and *W. globosa* [26, 27].

The new generations produced by vegetative propagation were called DFs or new buds (as in a budding yeast) and were released horizontally from the RpM. Usually, several individuals at different developmental stages coexisted in a single *W. globosa* frond. A single *W. australiana* frond produces 11 DFs on average and lives for about 17 days on average [16]. Each bud begins to senesce on the 10th day of survival [16]. Our study also confirmed the rapid propagation of *W. globosa* from the structural perspective. This reproductive strategy enabled rapid accumulation of biomass in *Wolffia*, which roughly doubled in 48 h, and allows its wide distribution in various habitats around the world [28]. The budding reproduction of *Wolffia* was clearly observed in our study by X-ray microtomography for the first time. *Wolffia* seldom flower under natural conditions, and no seed has been reported so far. However, *Wolffia* flowers have been reported in the laboratory [29, 30]; the causes of its reduction of sexual reproduction could be revealed in the future.

Our work and previous studies identified some differences in anatomical structure among different *Wolffia* species (Table 1). The most important was that the mesophyll cells of *W. arrhiza*, *W. borealis*, *W. australiana* and *W. globosa* showed a steep gradient in cell size from dorsum to ventrum with chloroplasts concentrated in the dorsal part, while *W. columbiana* had only a single mesophyll cell size and type with many fewer epidermal chloroplasts. Therefore, *W. columbiana* fronds would have a lower photosynthetic capacity compared with the other four *Wolffia* species [17]. From *Spirodela* to *Wolffia*, the size of fronds has changed; and the roots have been reduced, causing a reduction in the number of different cell types in *Wolffia* plants, which consist only of epidermis cells, mesophyll cells and some highly vacuolated cells. In contrast, *Spirodela* and *Lemna* plants have more cell types, especially vascular tissue [18]. The reduced body plan and size of *Wolffia* might be the reason for the presence of relatively few cell types in these plants. The evolution of Wolffia plants with higher specific surface area and individual flexibility also enabled them to acclimate well to changing environments. The morphology and structure of *W. microscopica* are also quite different from the above five *Wolffia* species. *W. microscopica* has a flattened frond and a presence pseudoroot, which did not exist in any other member of *Wolffia* [19]. These observations indicated that *W. microscopica* might be more closely related to duckweeds of other genera (*Spirodela*, *Lemna*, *Landoltia*) and thus link *Wolffia* and other duckweed plants [19]. Therefore, *W. microscopica* might be a key species in which to explore the basis of the observed root reduction in duckweeds.

**Table 1:** The anatomical structure of six *Wolffia* species

	*Wolffia arrhiza*	*Wolffia columbiana*	*Wolffia borealis*	*Wolffia australiana*	*Wolffia globosa*	*Wolffia microscopia*
Epidermis	A single epidermal layer with chloroplasts	A single epidermal layer with chloroplasts	A single epidermal layer with chloroplasts	A single epidermal layer with chloroplasts	A single epidermal layer with chloroplasts	The upper single epidermis almost completely lacks chloroplasts
Stomata	–	–	40–50	More than 40	314.34 ± 46.99 /mm^2^	Two to three rows of stomata in the dorsal surface
Mesophyll	The upper palisade-like cells subjacent to the lower vacuolated cells	A single mesophyll cell size and type	Vary in size from small cells toward the dorsum to large cells toward ventrum	The upper third was loosely packed palisade-like parenchyma cells, the lower two thirds were composed of large, highly vacuolated cells	Vary in size from small and dense palisade-like parenchyma cells in dorsum to large and empty vacuolated cells in ventrum	Centre composed of loosely arranged parenchymatic cells interspersed with air spaces towards the dorsal side and more tightly packed parenchymatous cells with small air spaces towards the ventral surface
Chloroplasts	Concentrated in epidermis, mesophyll cells and vaculated cells	Mainly concentrated in epidermis	Concentrated in dorsal mesophyll cells	Concentrated in dorsal mesophyll cells	Both in the mesophyll cells and epidermis	Prominent in the lower epidermis and ventral parenchymatic cell layers
Transfer cells	–	Present in the epidermis	Absent	–	Present in adjacent mature mesophyll cell	–
Pseudoroot	Absent	Absent	Absent	Absent	Absent	Present
Reference	[15]	[17]	[17]; [18]	[16]	Current study	[19]

The reduction of the root in *Wolffia* is one of the most striking examples of structural reduction in the plant kingdom. Duckweeds include five genera; members of *Wolffia* and *Wolffiella* are rootless, and members of *Spirodela*, *Landoltia*, and *Lemna* produce either a single or few roots [31]. There is a reduction of the number of roots from *Spirodela* to *Lemna* and they disappear entirely in *Wolffia*. Early studies suggested that duckweeds did not use their roots to acquire nutrients, and instead acquire nutrients through their fronds [32–35]. Echlin et al. [36] found that most absorption of ions occurred in the root tip region of *L. minor*, and observed a Casparian band structure in the endodermis of the root tip. They suggested that the root of *L. minor* can not only absorb nutrients and water but also transport these to the frond. Kim [37] carried out a detailed study of root development of *S. polyrhiza* and found a large number of plasmodesmata between the cells of the root. They therefore concluded that the transport of metabolites between the root and frond may rely on the symplastic pathway. Cedergreen and Madsen (2003) reported that both the root and fronds of *L. minor* had the ability to absorb NO_3_^−^ and this ability was affected by light irradiance. The root of Fand stabilize the plant on the water surface. However, White and Wise [17] suggested the rootless *Wolffia* stay afloat and upright not by buoyancy but by surface tension. In their opinion, if buoyancy kept *Wolffia* plants at the water’s surface, then they would sink late in the day as their starch content reached a maximum. In our study, the dorsal part of *W. globosa* was always above the water, and it was difficult to submerge the plants or turn them over. In addition, most of the chloroplasts, which were filled with starch grains, were concentrated at the dorsal side. Previous studies have shown that dormant individuals of *Wolffia* were full of starch grains and sank in the water [20]. We speculate that the content of starch grains affects the stable floating of *Wolffia*. Furthermore, the loss of the nutrient uptake and stabilization functions of the root in *Wolffia* may have allowed them to lose this organ. Phylogenetic analysis using different molecular markers has confirmed that duckweeds comprise a single monophyletic clade [38], suggesting that rootlessness has a single evolutionary origin in Lemnoideae.

We propose that *Wolffia* is a suitable model to study structural reduction in angiosperms and to explore the cause of rootlessness. First, *Wolffia* is easy to cultivate, completes its life cycle in the lab, and reproduces quickly. Second, *Wolffia* plants can be genetically transformed, as can the rooted *Spirodela* and *Lemna*, allowing us to conduct genetic studies [21, 39, 40]. *Wolffia* species are the smallest flowering plants in the world, in both size and morphological structures, containing one leaf, one stamen and one gynoecium, which represent the core elements for angiosperms to complete their life cycle. Hillman [41] pointed out that although the gross morphology and vegetative reproduction of *Lemnaceae* are somewhat unusual, their anatomy, particularly the prominent air spaces and reduced vascular structures, resembles that of many aquatic angiosperms. Anderson et al. [15] also pointed out that although *Wolffia* lacks vascular tissue, the range of tissue and cell types appears as heterogeneous as in most leaves and varies considerably from meristematic to mature chlorenchymous tissue. Not only is it suitable to study structural reduction, but *Wolffia* would also be an ideal system to explore fundamental processes of angiosperm development that cannot be addressed using other model plants.

## Conclusions

This first comprehensive study of the morphology and anatomy of *W. globosa.* Revealed that the morphology of *W. globosa* did not fit the traditional botanical descriptions. The rootless *W. globosa* budded new fronds from the unique meristematic area by vegetative propagation, and usually several generations coexisted in a single frond, as observed in other *Wolffia* species*.* This reproductive strategy enabled rapid accumulation of biomass and their wide distribution in various habitats around the world. The reduced body plan and size of *Wolffia* might be the reason for the presence of relatively few cell types in these plants. We also propose that *Wolffia* plants are not only suitable for the study of structural reduction in higher plants, but also an ideal system to explore the fundamental developmental processes of higher plants that cannot be addressed using other model plants.

## Methods

### Plants cultivation and identification

*W. globosa* (5563) plants were collected from East Lake (N30°32′, E114°21′) at the city of Wuhan, Hubei Province, China (no permission was required to collect such plant samples). Plants were sterilized in 0.1% mercuric chloride for 2–3 min and then cultured in half-strength (1/2) Schenk & Hildebrandt (SH) medium [42] at pH 5.5 containing 1% (w/v) sucrose and 0.8% (w/v) agar. Regenerated fronds of *W. globosa* were transferred to liquid 1/2 SH medium for longer preservation. Cultivation was conducted at 25 ± 1 °C under white light of 85 μmol m^− 2^ s^− 1^ and 16-h day/8-h night photoperiod. *W. globosa* fronds in good condition were selected for experiments.

The identification of *W. globosa* (5563) was conducted by Jingjing Yang and P.P.M. Heenatigala using *atpF-atpH* barcode primers [21, 43]. The identification results were submitted to the Rutgers Duckweed Stock Cooperative at the State University of New Jersey (http://www.ruduckweed.org/register.html). *W. globosa* (5563) plants were preserved at the National Aquatic Biological Resource Center.

### 3D structure observation of *W. globosa* frond by X-ray microtomography (MicroCT) imaging

We first used MicroCT to explore the morphology and internal structure of the *W. globosa* frond. The fronds were scanned at the MicroCT facility (Skyscan1267, Burker) and scans were obtained at a spatial resolution of 3 μm (4032 × 2688 pixel field of view), with an electron acceleration energy of 85 kV and a current of 100 μA. Detector exposure time was 750 ms, collecting 412 projections in “step and shoot” mode with no averaging, resulting in a scan duration of 9 min per sample. Radiograph reconstruction was carried out using NRecon reconstruction software (version 1.7.4.2, Bruker) with a beam hardening correction of 15. Finally, the scanned area beyond the plant sample was removed and reconstructed into 3D volumes using a filtered back-projection algorithm.

### Light and electron microscope observation

For SEM, the fresh fronds were fixed in 2.5% glutaraldehyde in phosphate-buffered saline (PBS) buffer (1 M, pH 7.4) overnight at 4 °C followed by a stepwise ethanol and tert butanol dehydration. Then samples were dried using a freeze dryer (Hitachi ES-2030). The obtained specimens were examined with a scanning electron microscope (Hitachi S4800) at 30 kV.

For light and transmission electron microscopy (TEM), the samples were washed in PBS buffer after fixing overnight at 4 °C. Then samples were post-fixed with 1% OsO_4_ in PBS for 2 h at 4 °C following stepwise ethanol and acetone dehydration and infiltration with Spurr’s epoxy resin. The treated samples were embedded and polymerized in Spurr’s epoxy resin at 60 °C for 48 h. Sections for light microscopy were cut using a LEICA EM UC 7 instrument with a glass knife and stained with 1% toluidine blue. The obtained specimens were photographed with an OLYMPUS BX53 camera. Ultra-thin sections (70 nm) for TEM were also cut using a LEICA EM UC 7 instrument and double-stained with 2% uranyl acetate and Sato’s lead citrate. The obtained specimens were examined with a transmission electron microscope (Hitachi-7700) at 120 kV.

## Supplementary Information

**Additional file 1: Movis S1**. The three-dimensional volumes of *W. globosa* frond.

**Additional file 2: **The raw data of **Fig.****1**.

**Additional file 3: **The raw data of **Fig.****2**.

**Additional file 4: **The raw data of **Fig.****3**.

**Additional file 5: **The raw data of **Fig.****4**.

## Data Availability

All data generated or analyzed during this study are included in this article (and its supplementary files) or available from the corresponding author on reasonable request. Plant materials are available from the corresponding author.

## Abbreviations

3D: Three-dimensional
MicroCT: X-ray microtomography
PBS: Phosphate-buffered saline
RAM: Root apical meristem
SAM: Shoot apical meristem
SEM: Scanning electron microscopy
TEM: Transmission electron microscopy
