# Supplementary material for: Frond architecture of the rootless duckweed Wolffia globosa
Source: BMC Plant Biol. 2021 Aug 20;21:387. doi: 10.1186/s12870-021-03165-5 (PMC8377843; doi:10.1186/s12870-021-03165-5)

## Slide 1
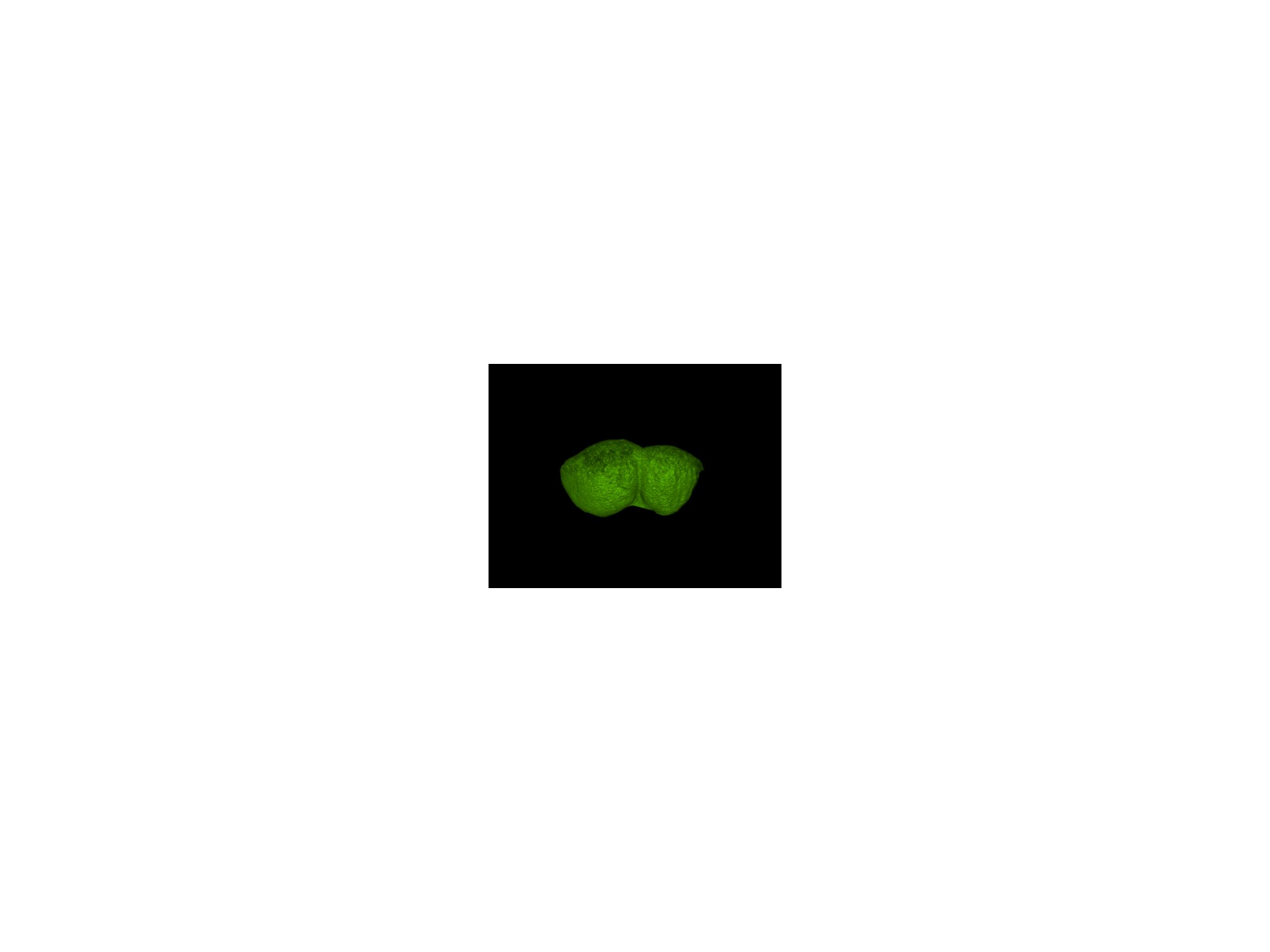

## Slide 2
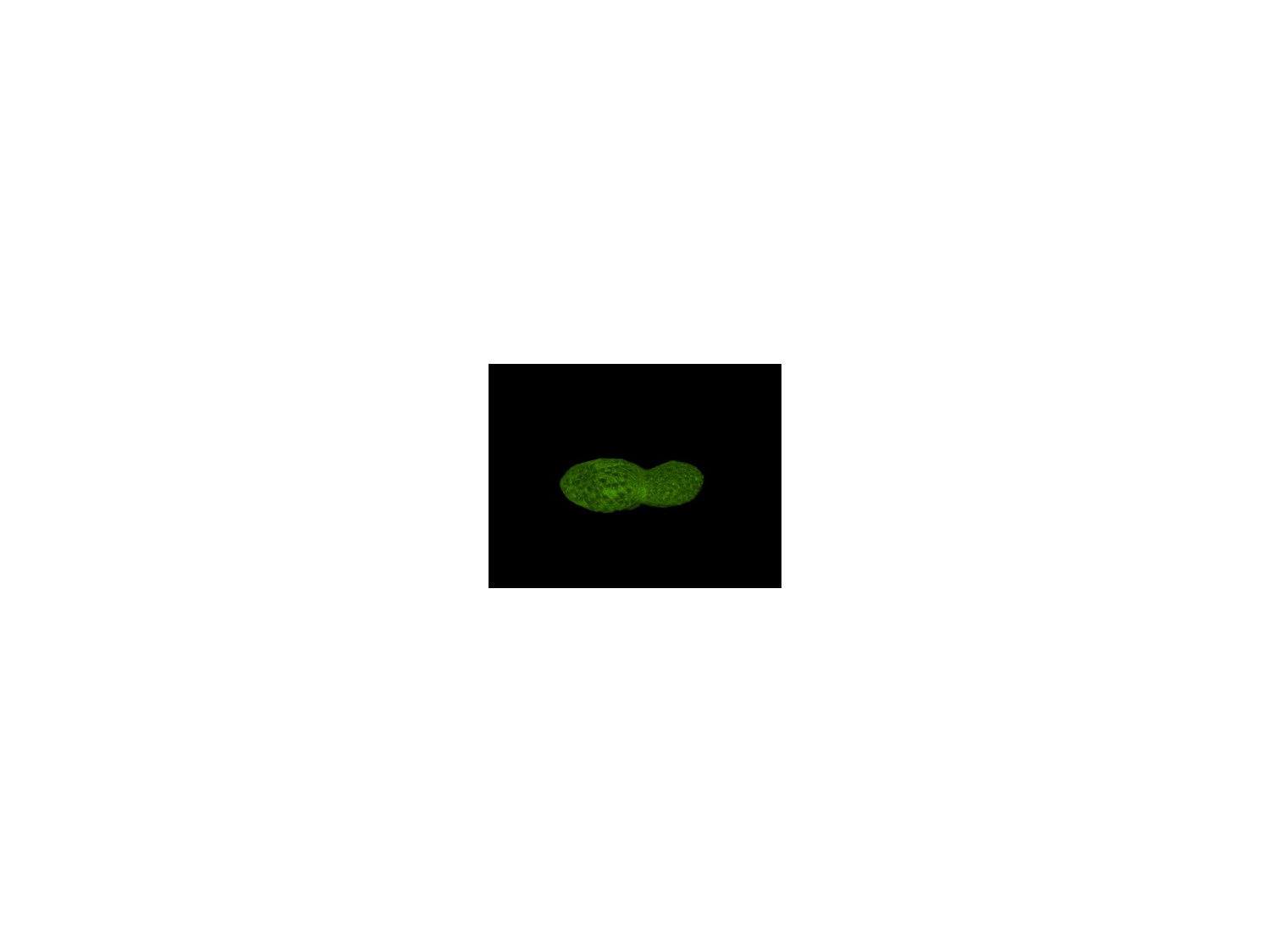

## Slide 3
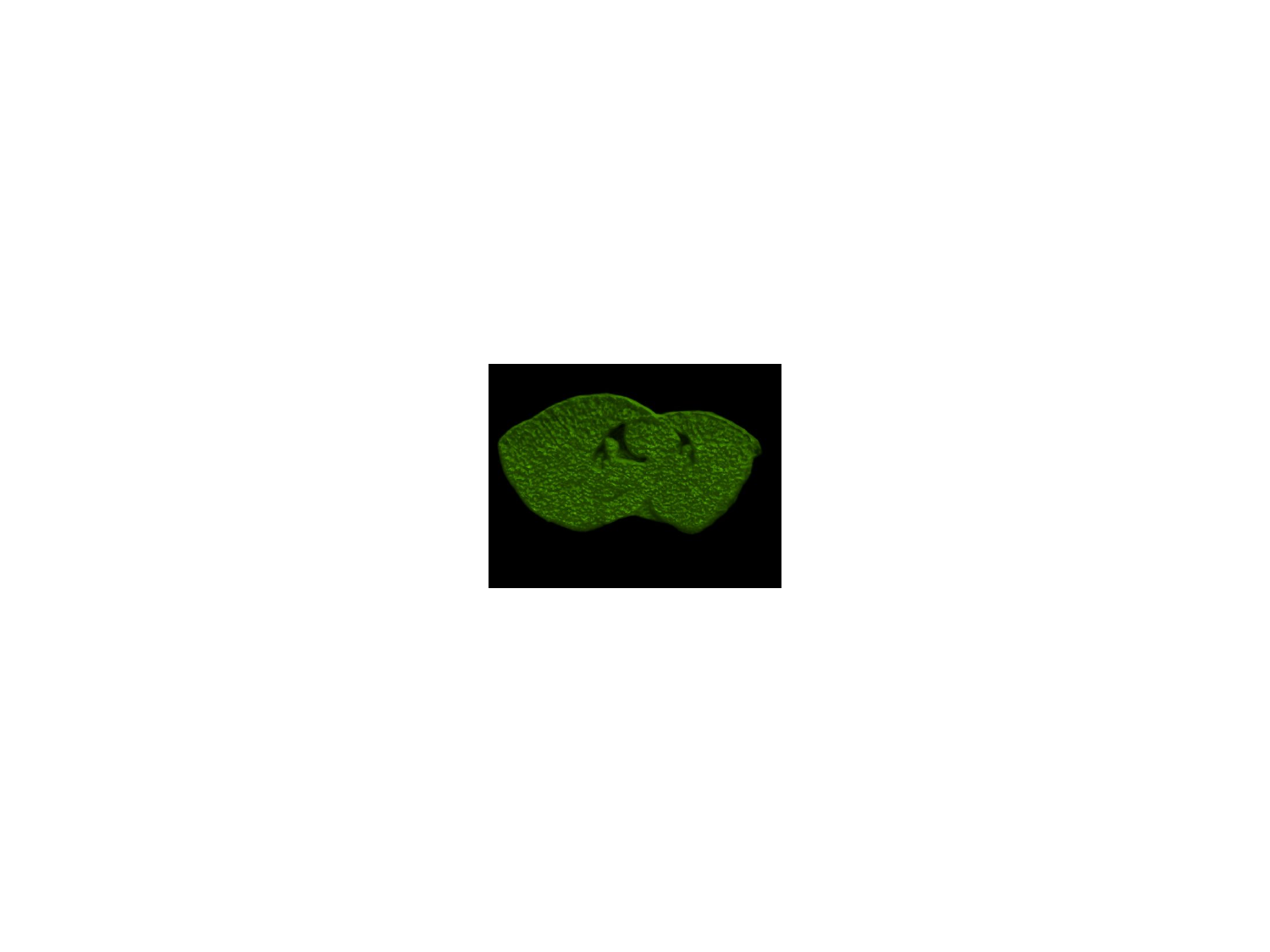

## Slide 4
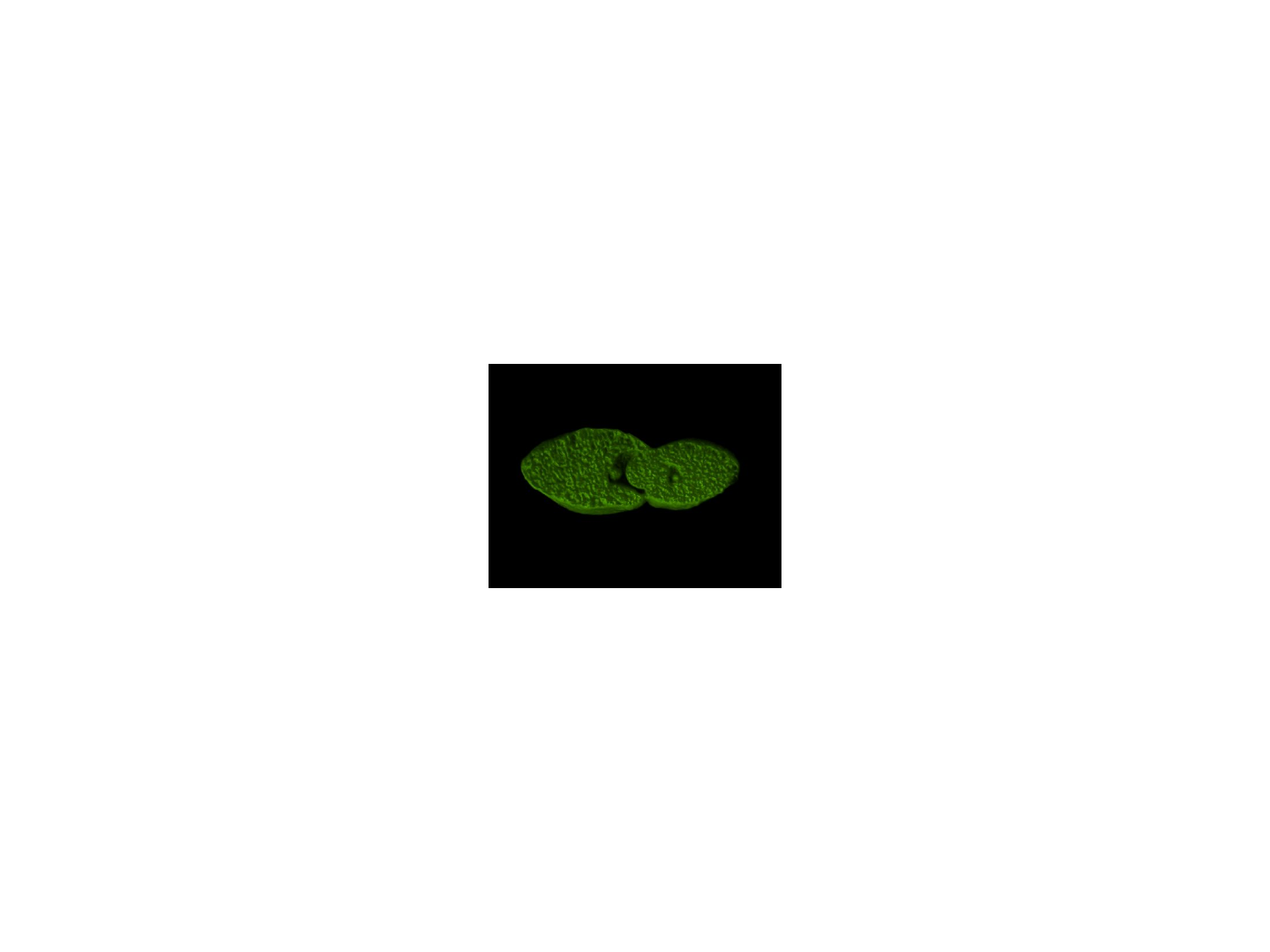

## Slide 5
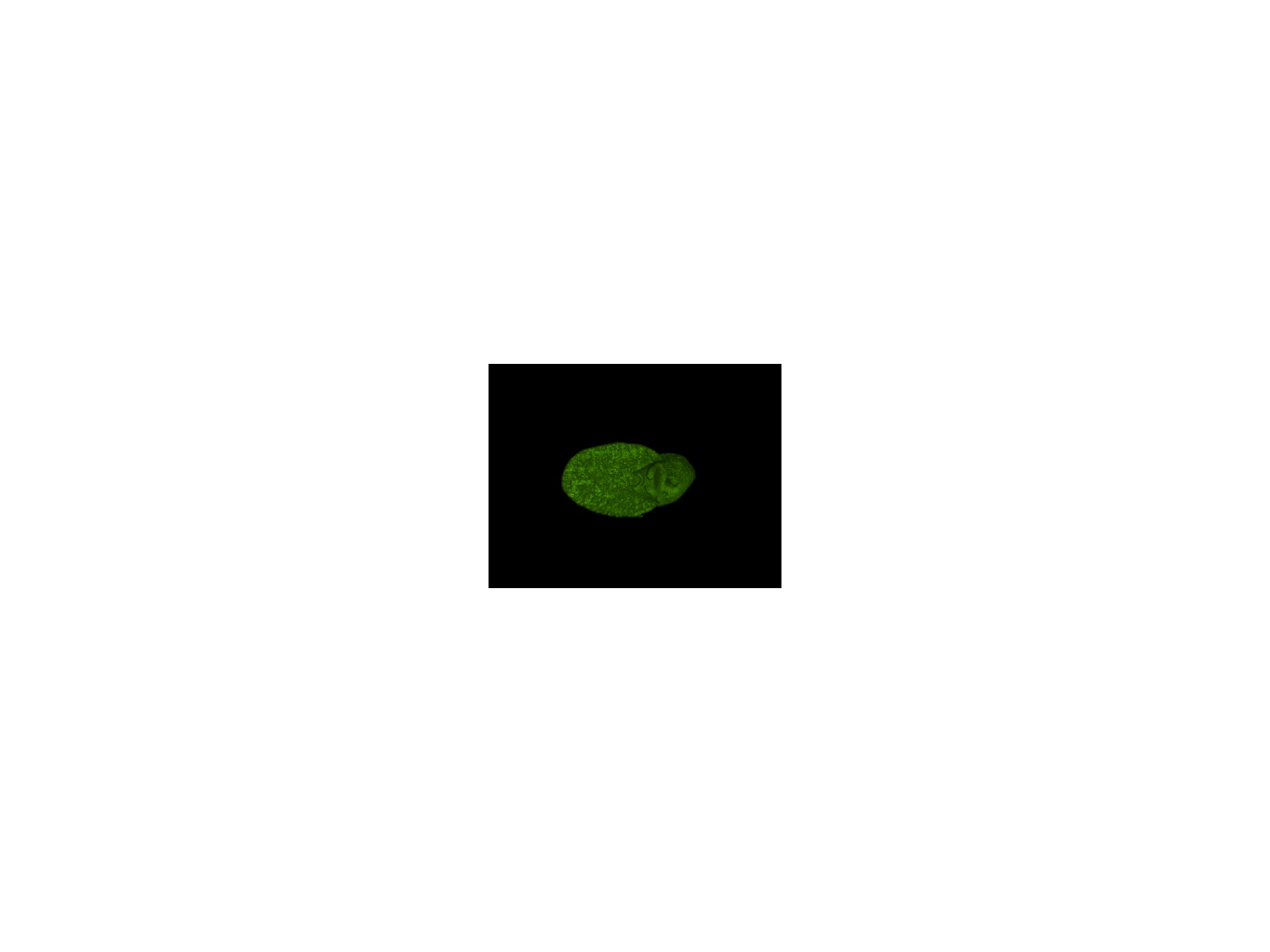

## Slide 6
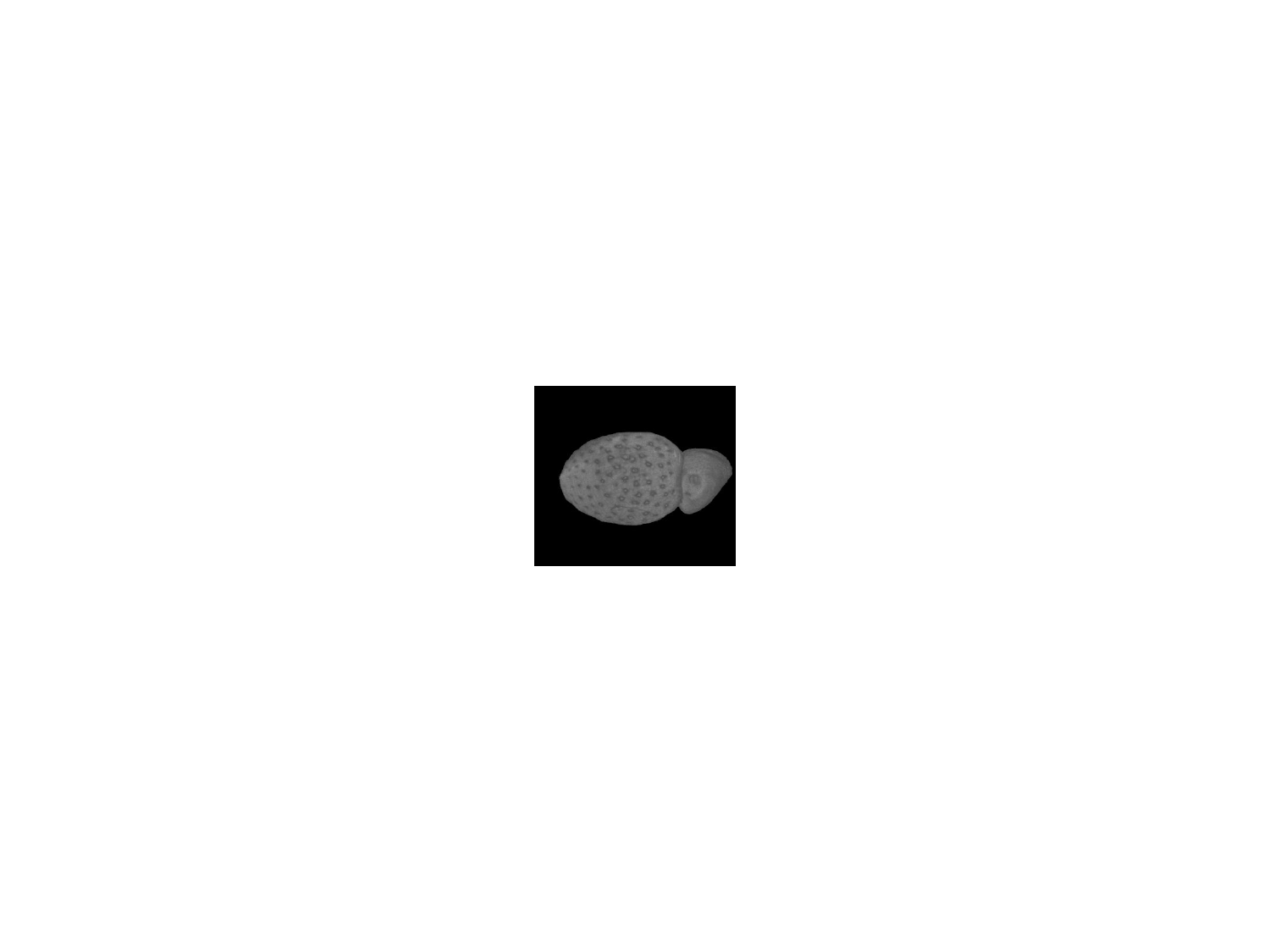

## Slide 7
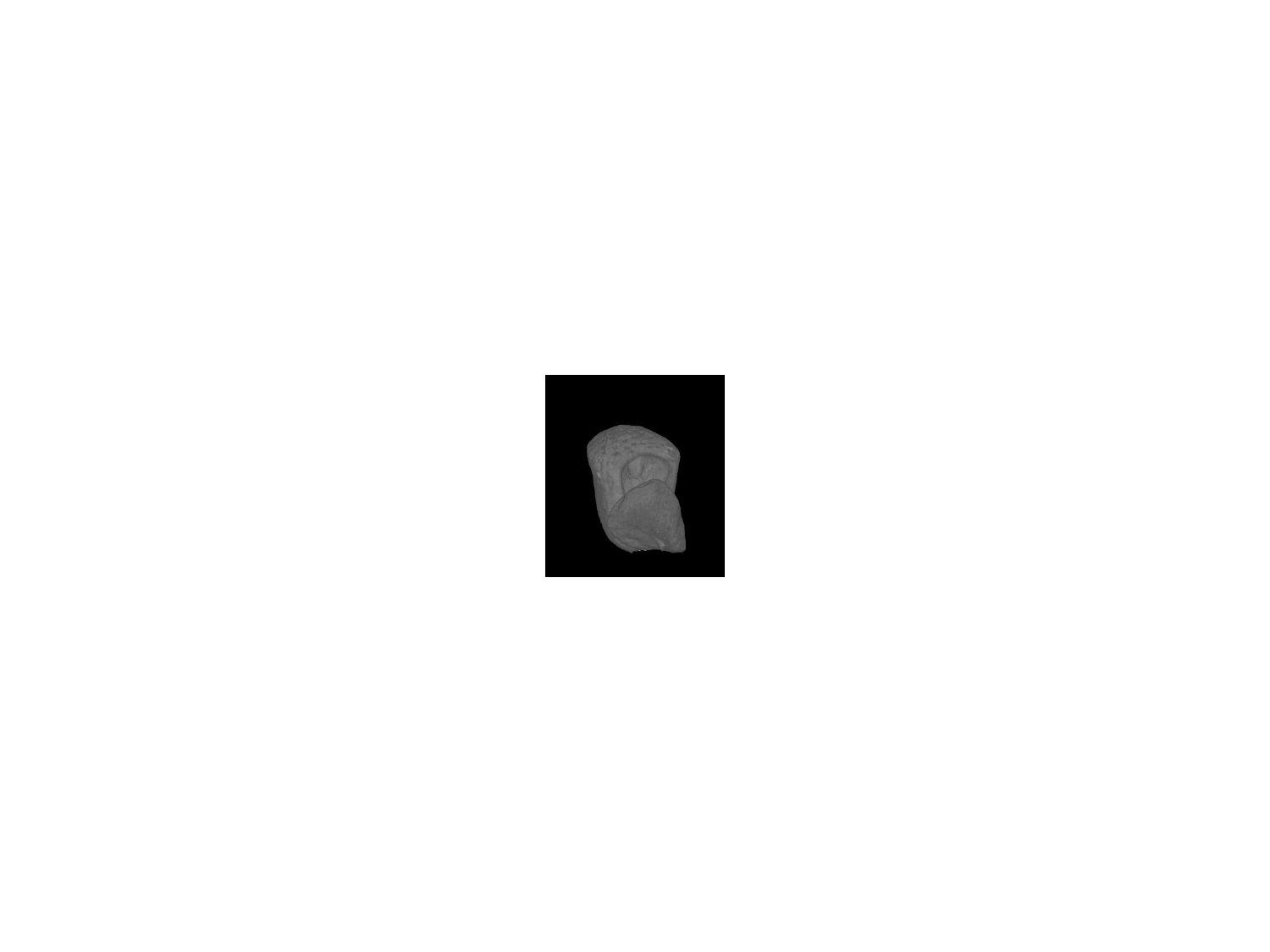

## Slide 8
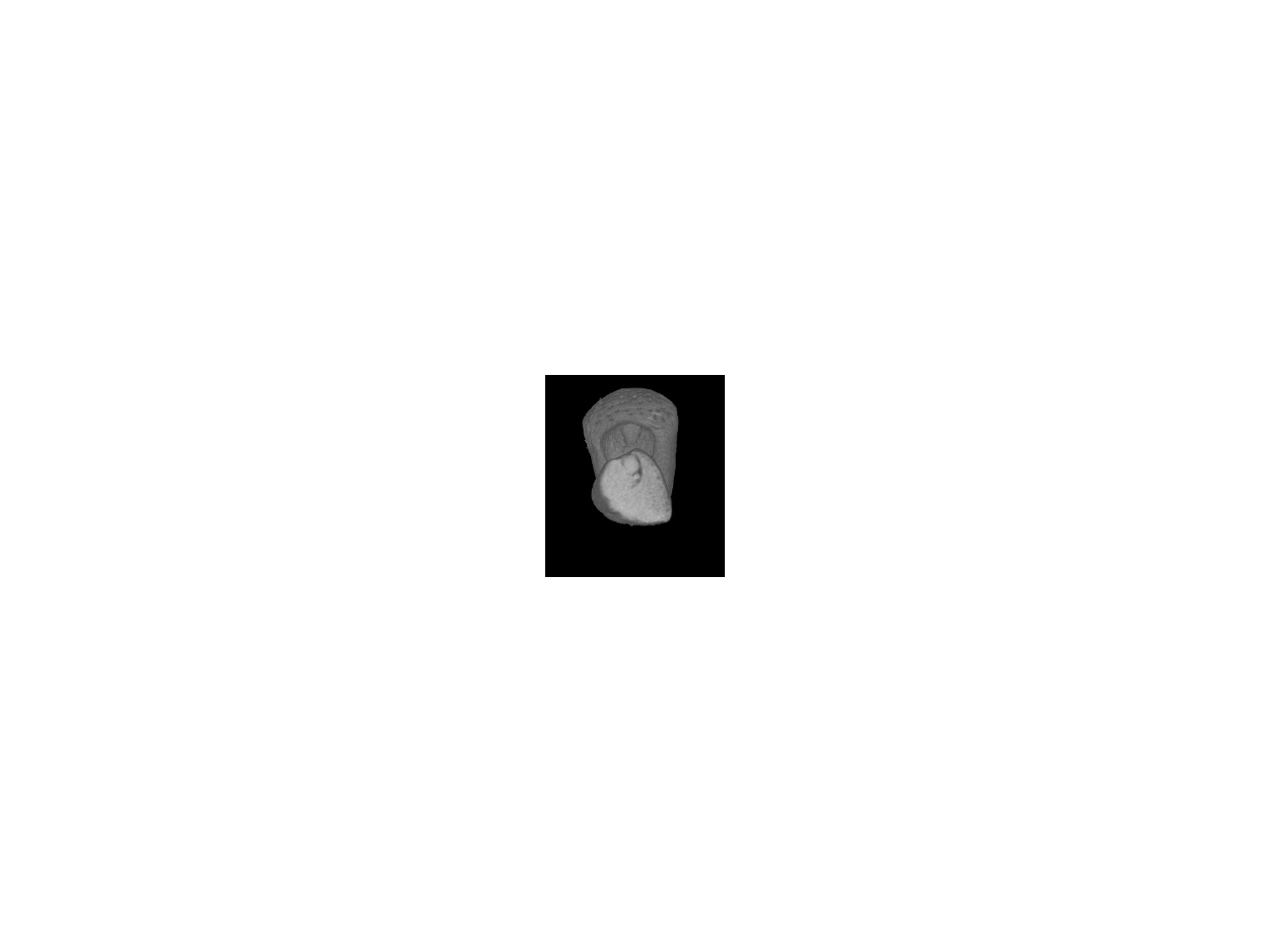

## Slide 9
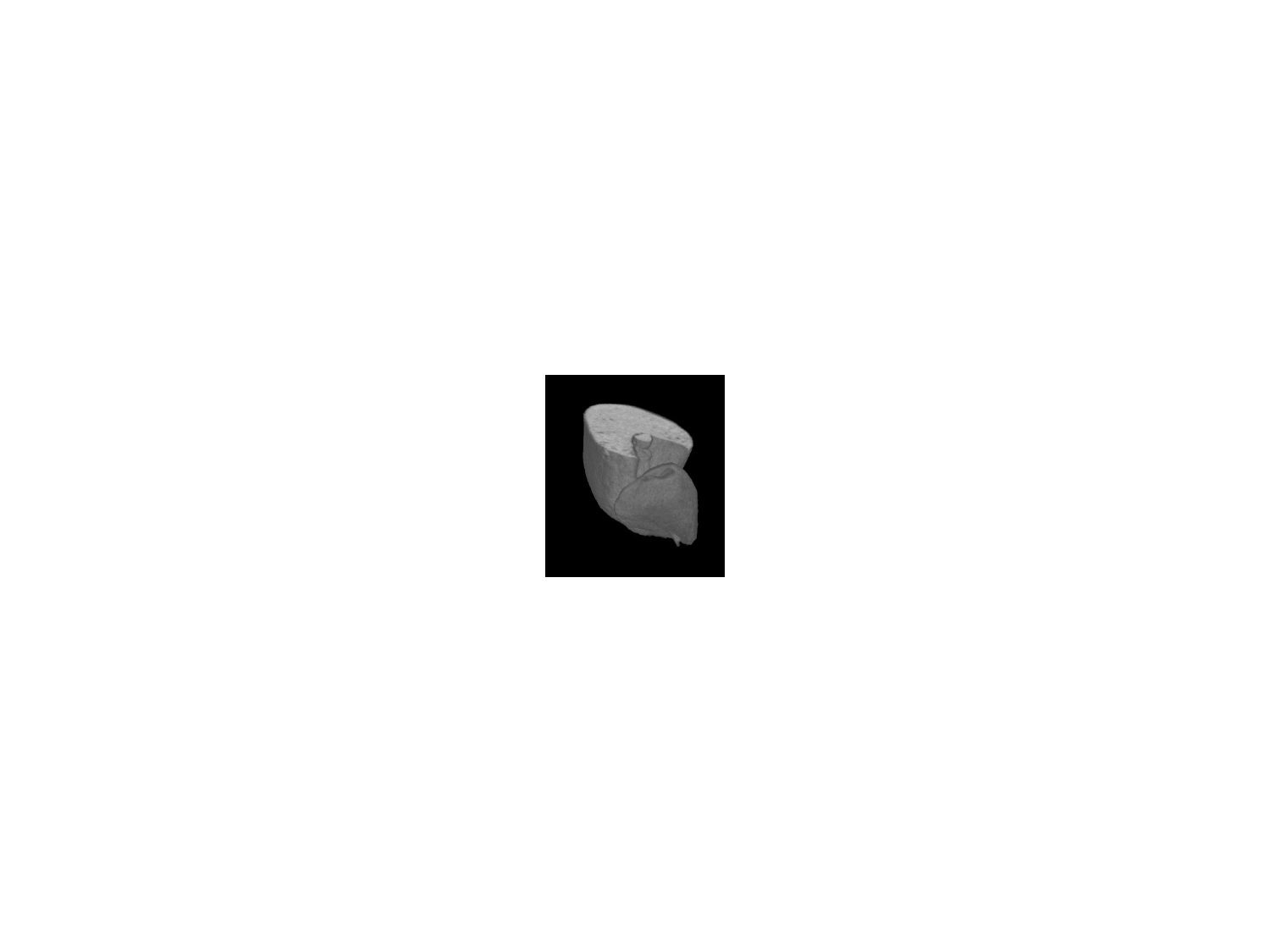

## Slide 10
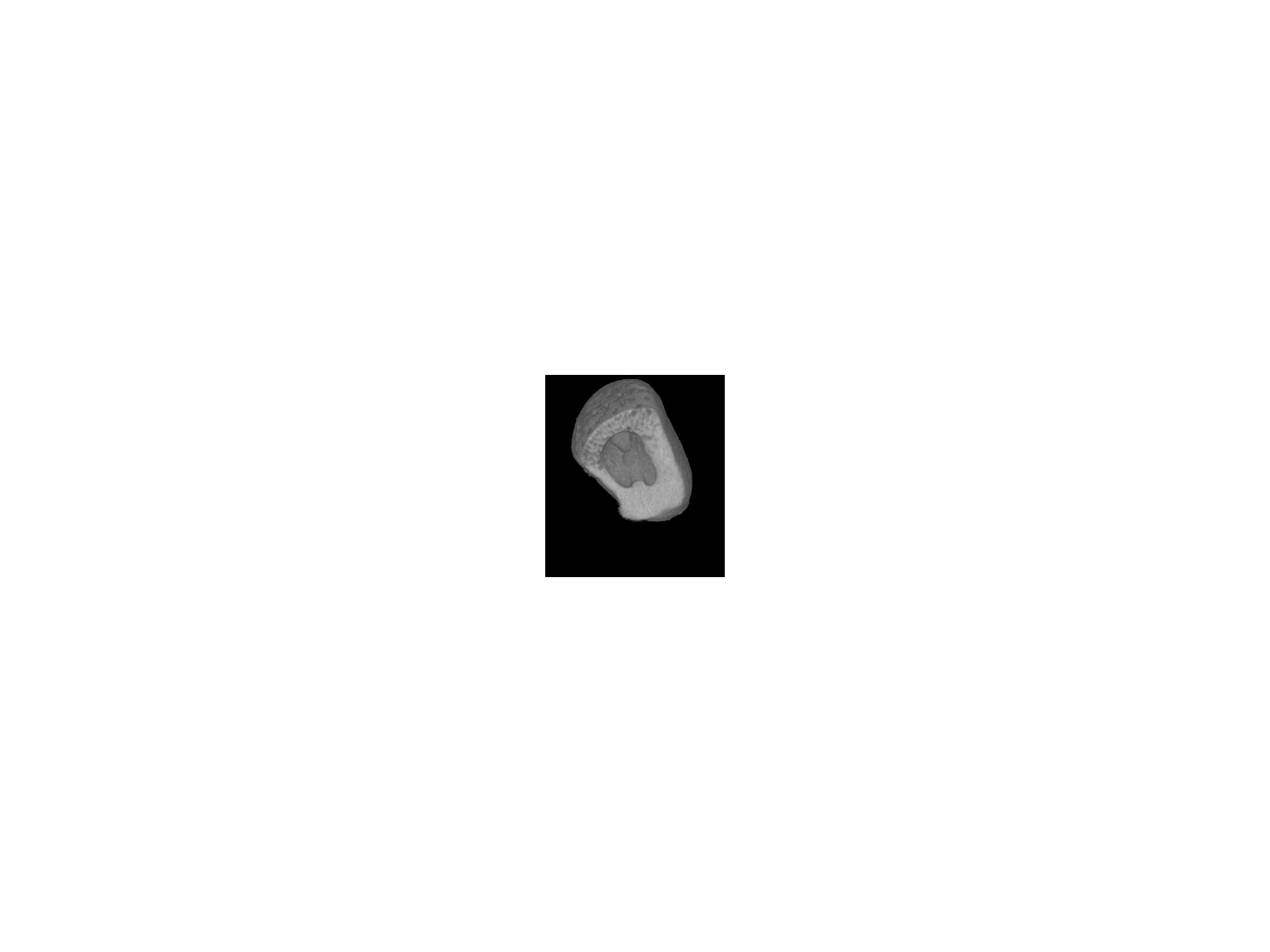

## Slide 11
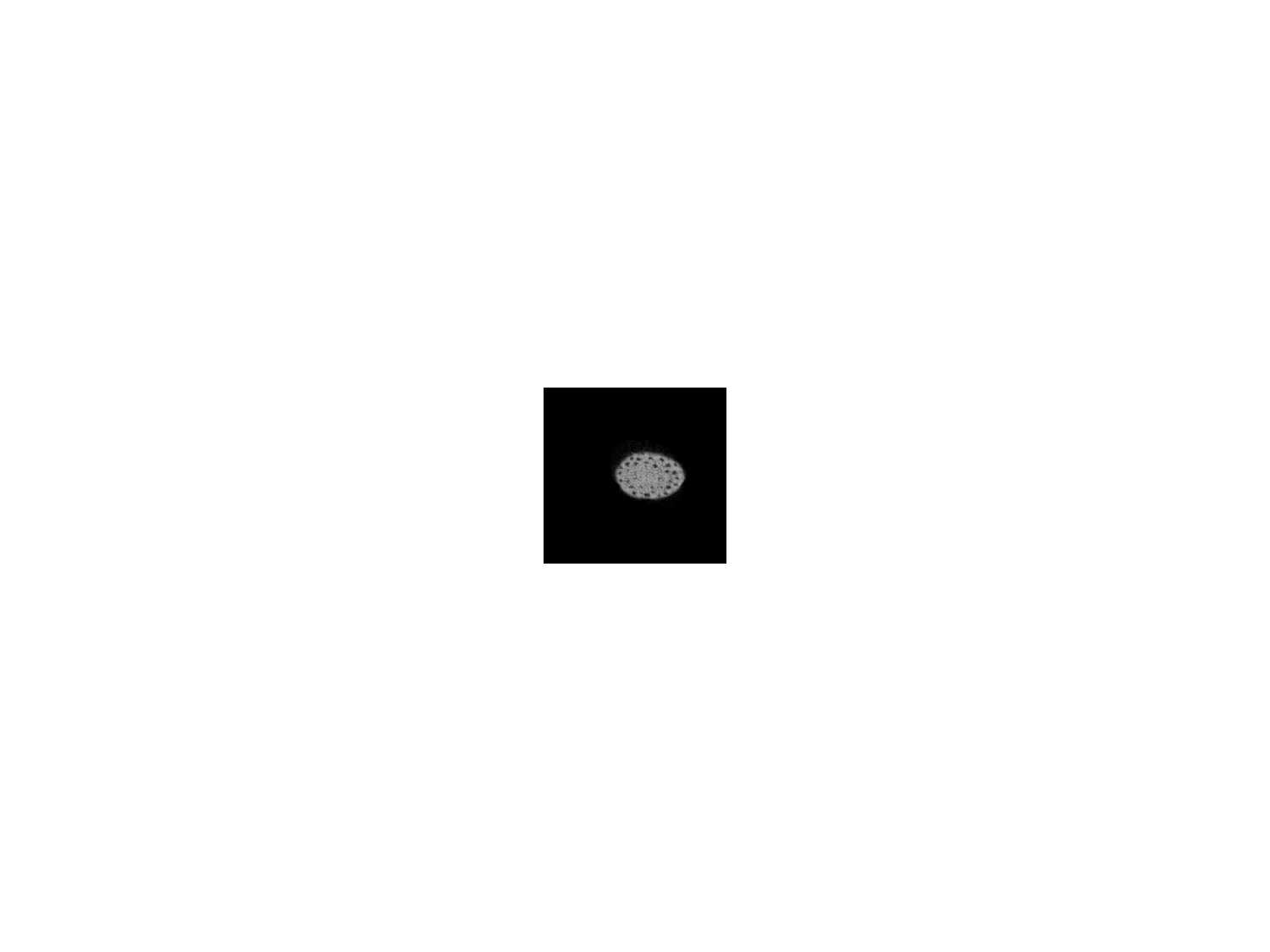

## Slide 12
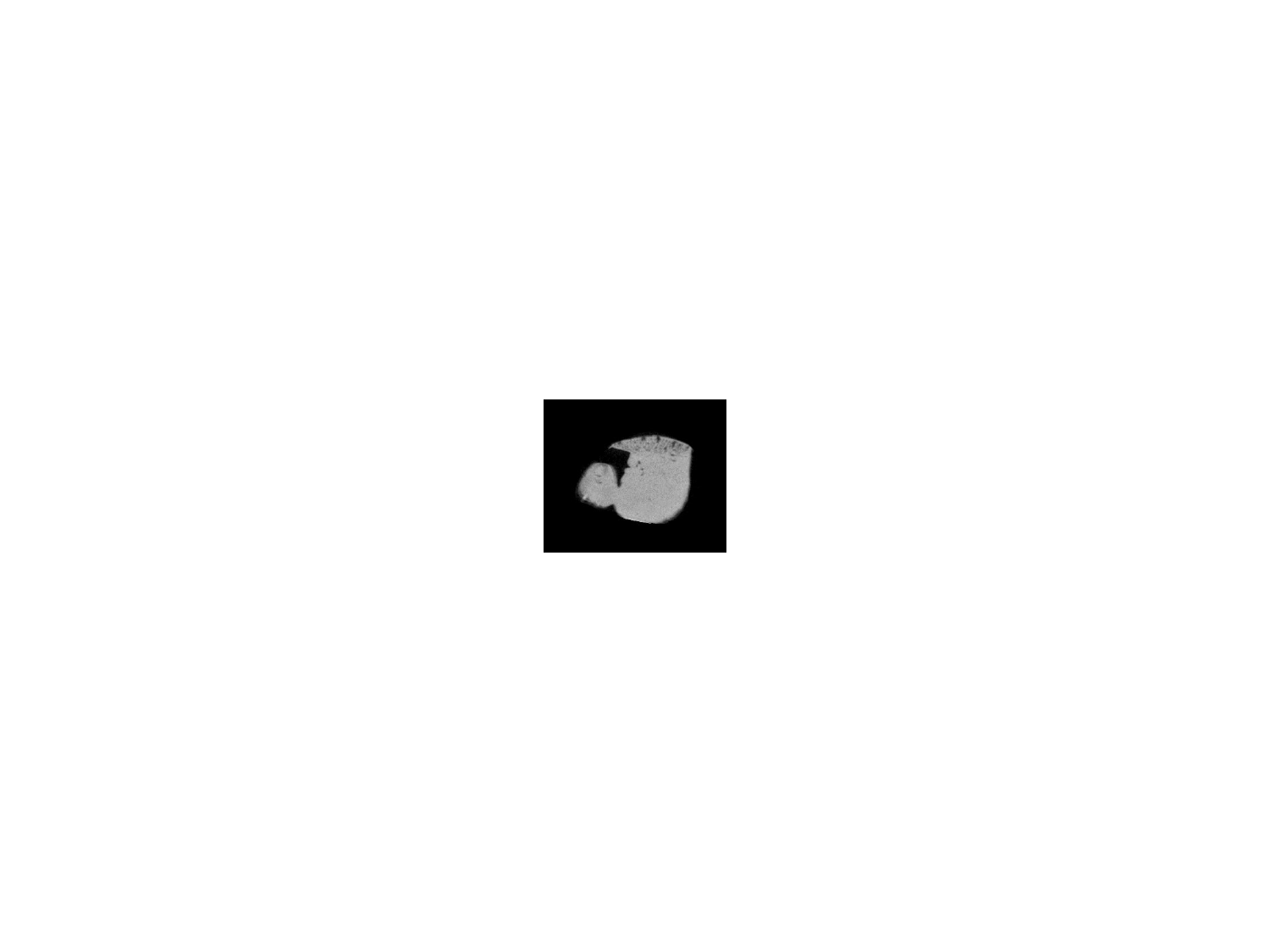

## Slide 13
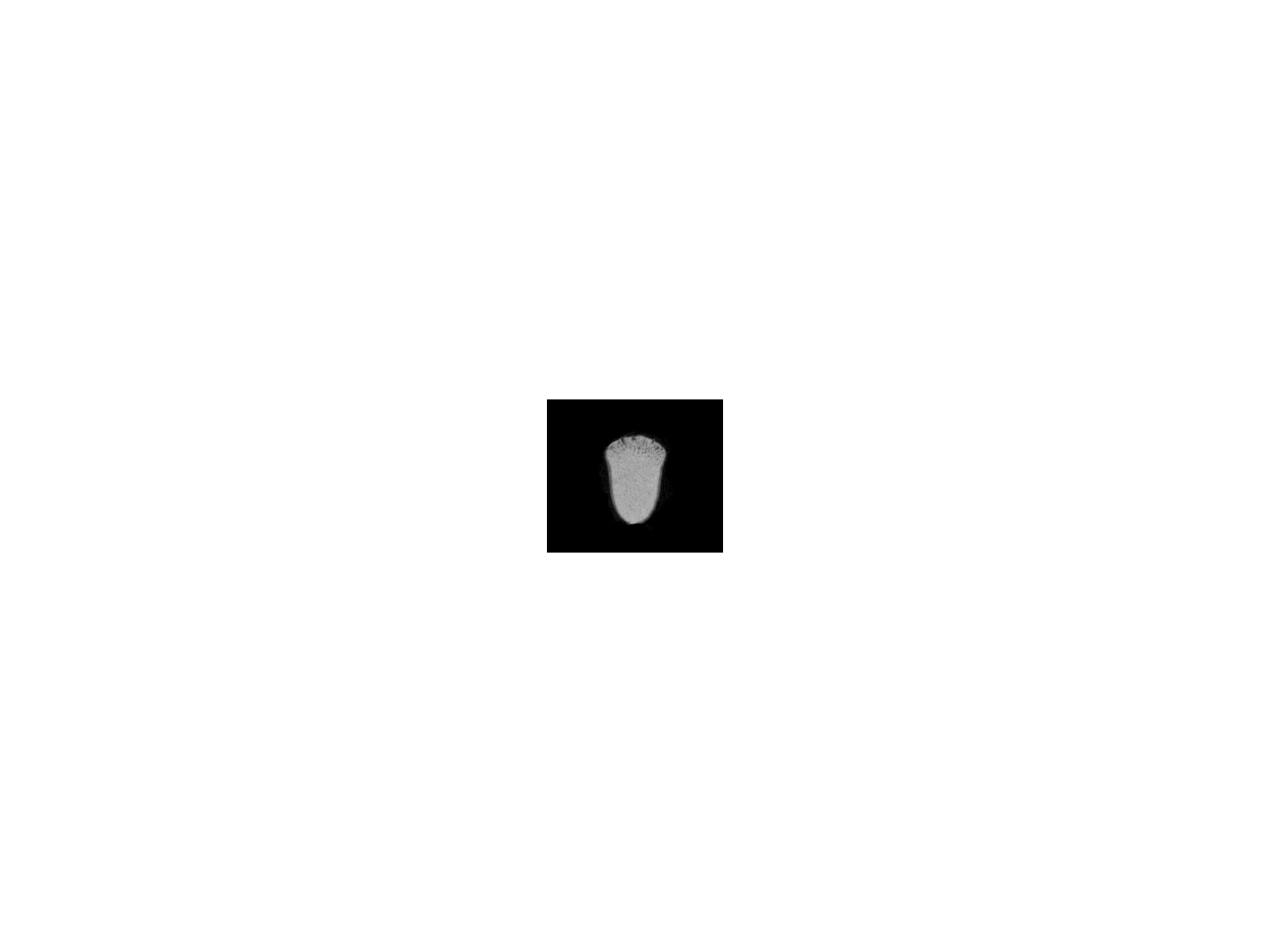

## Slide 14
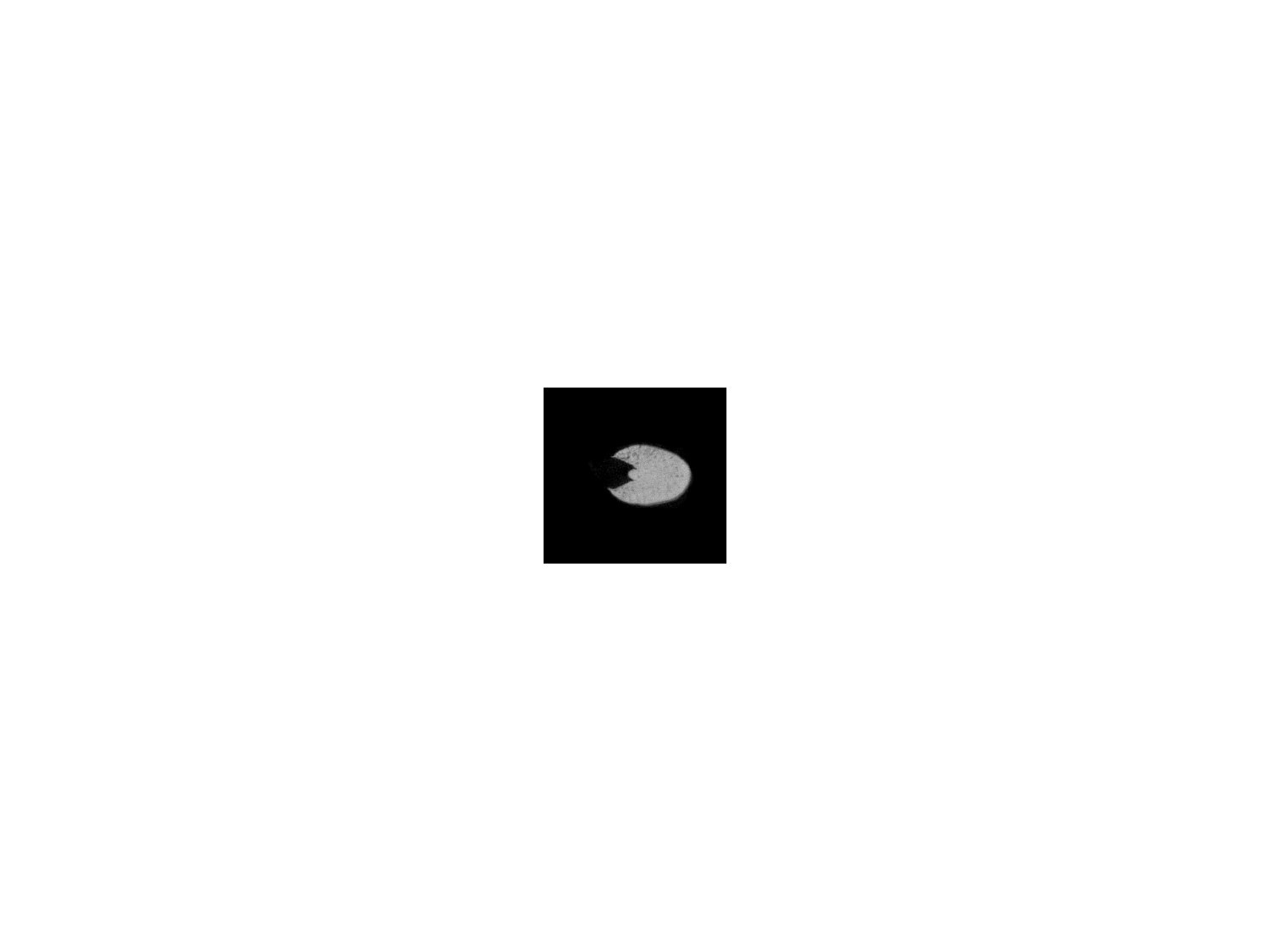

## Slide 15
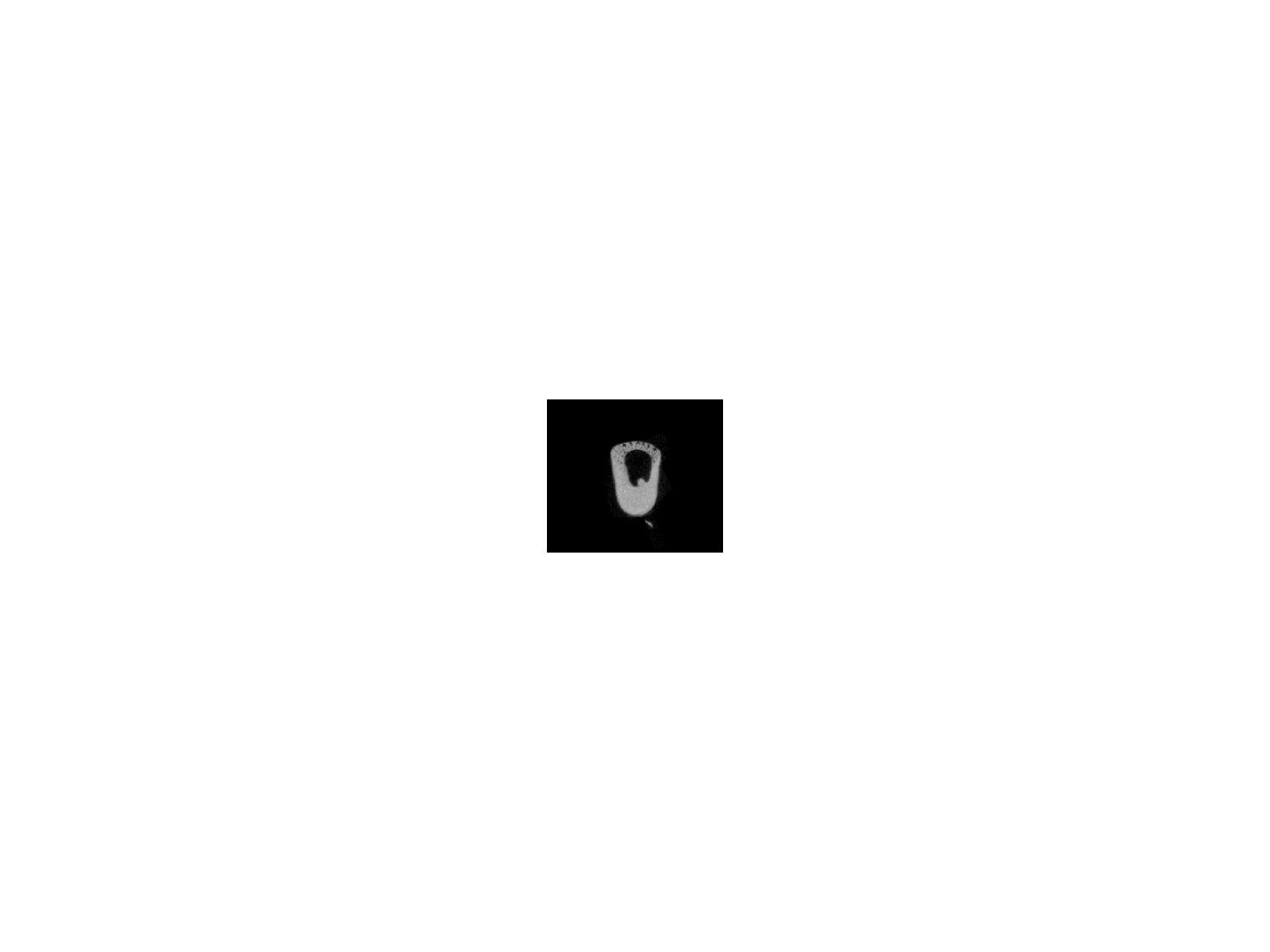

## Slide 16
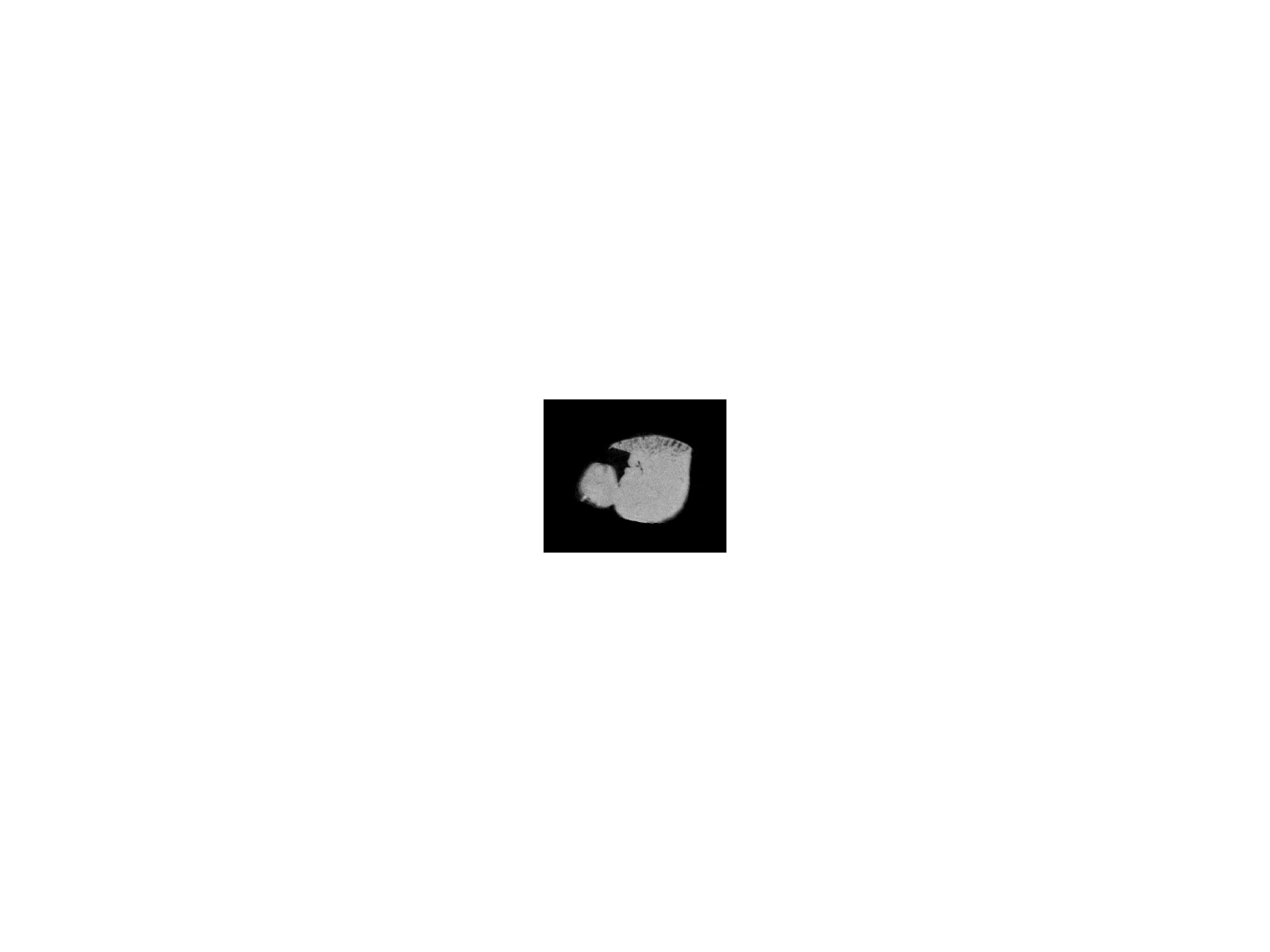

Supplement: Supplementary file 2 — Additional file 2: The raw data of Fig.1. [file 12870_2021_3165_MOESM2_ESM.pptx]
