# Supplementary figures and images for: Frond architecture of the rootless duckweed Wolffia globosa
Source: BMC Plant Biol. 2021 Aug 20;21:387. doi: 10.1186/s12870-021-03165-5 (PMC8377843; doi:10.1186/s12870-021-03165-5)

## Slide 1
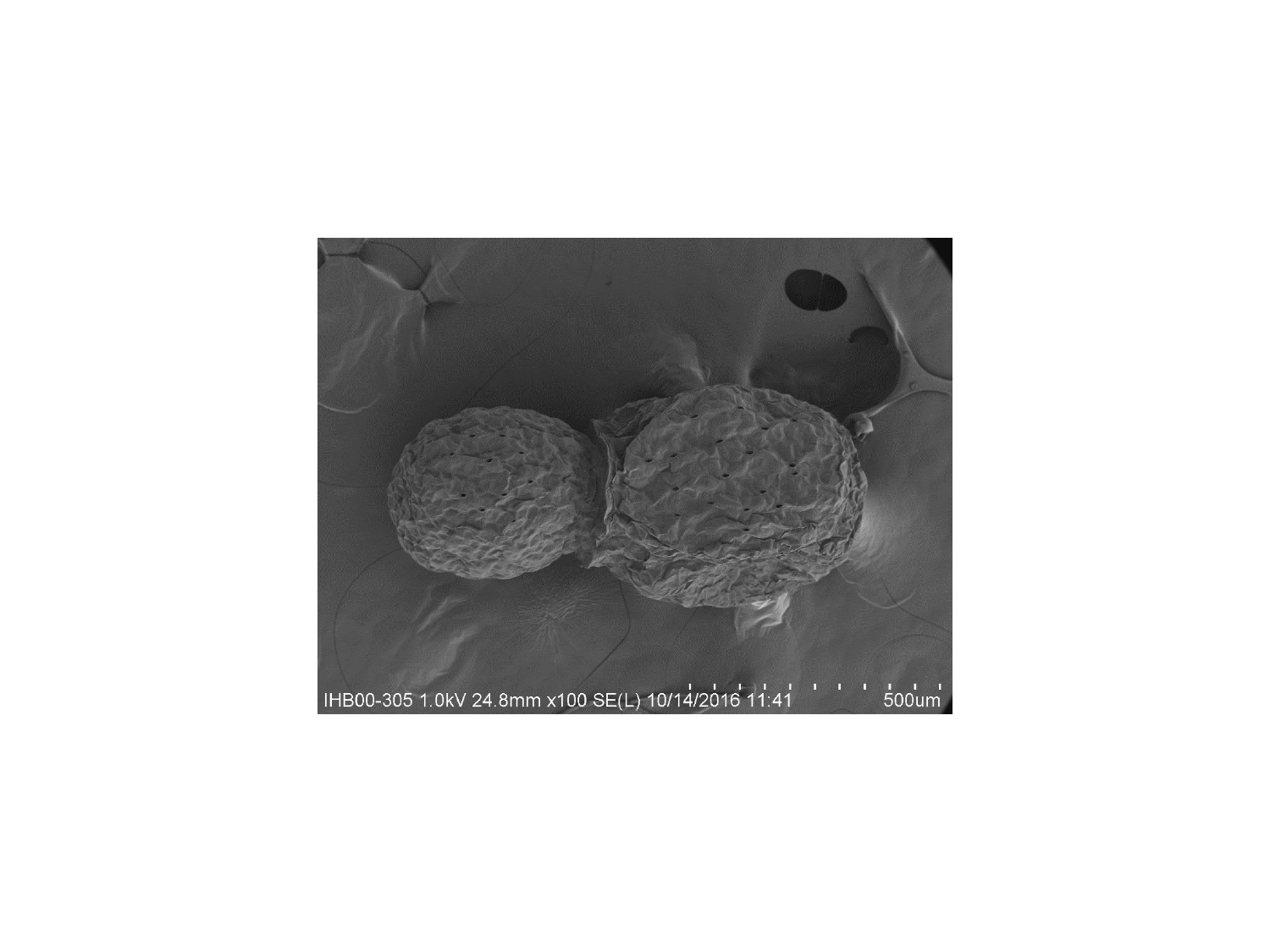

## Slide 2
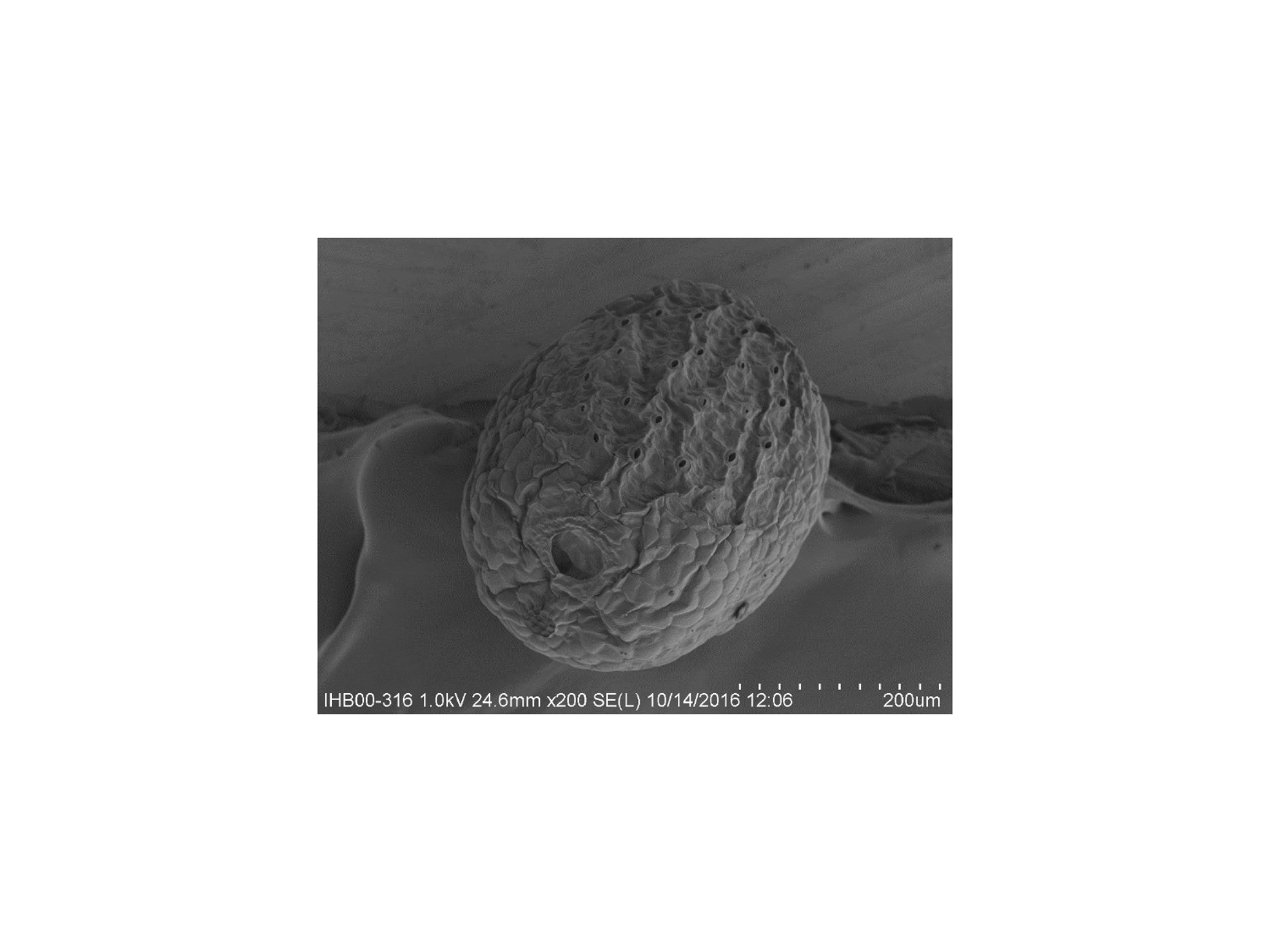

## Slide 3
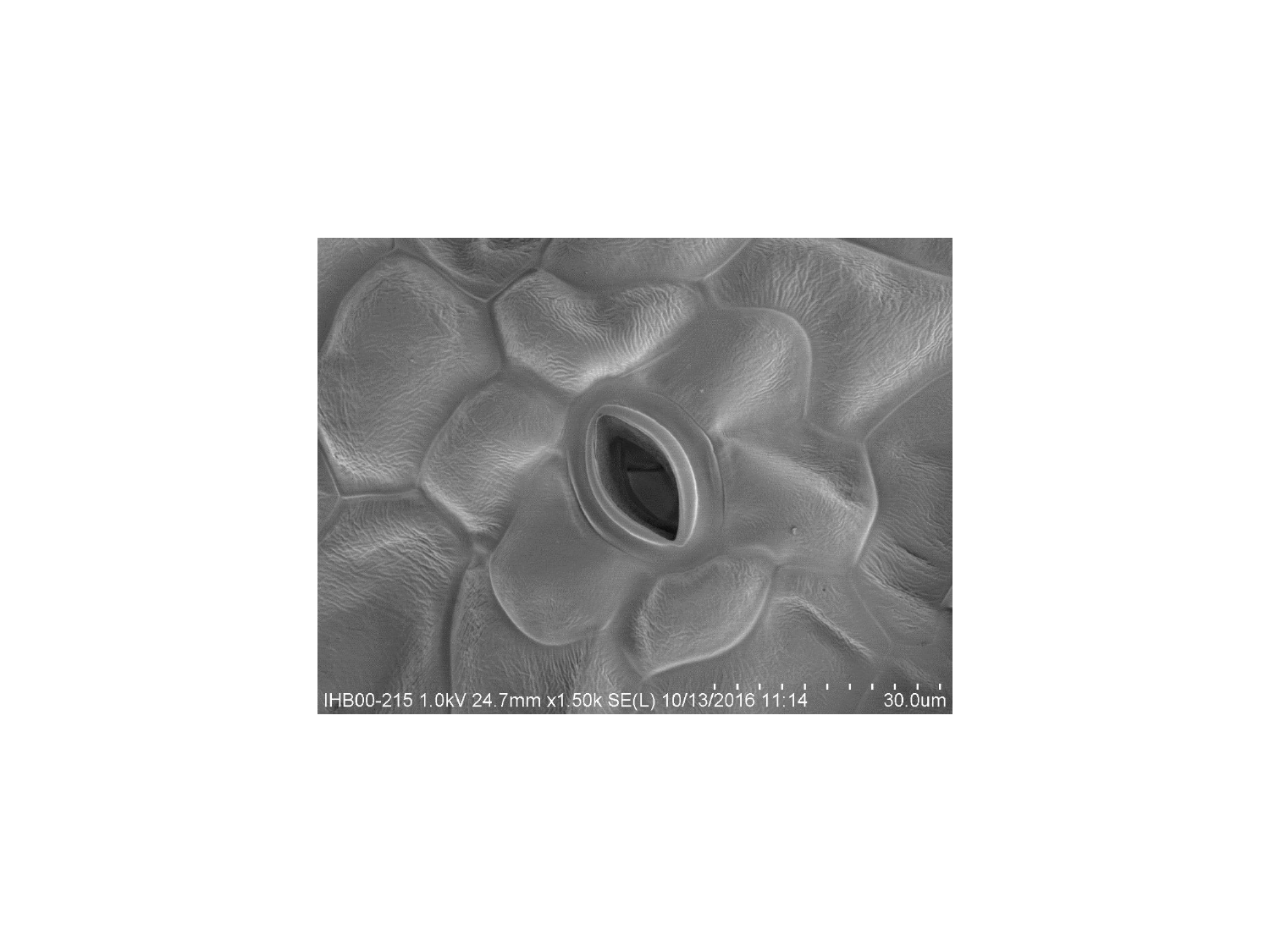

## Slide 4
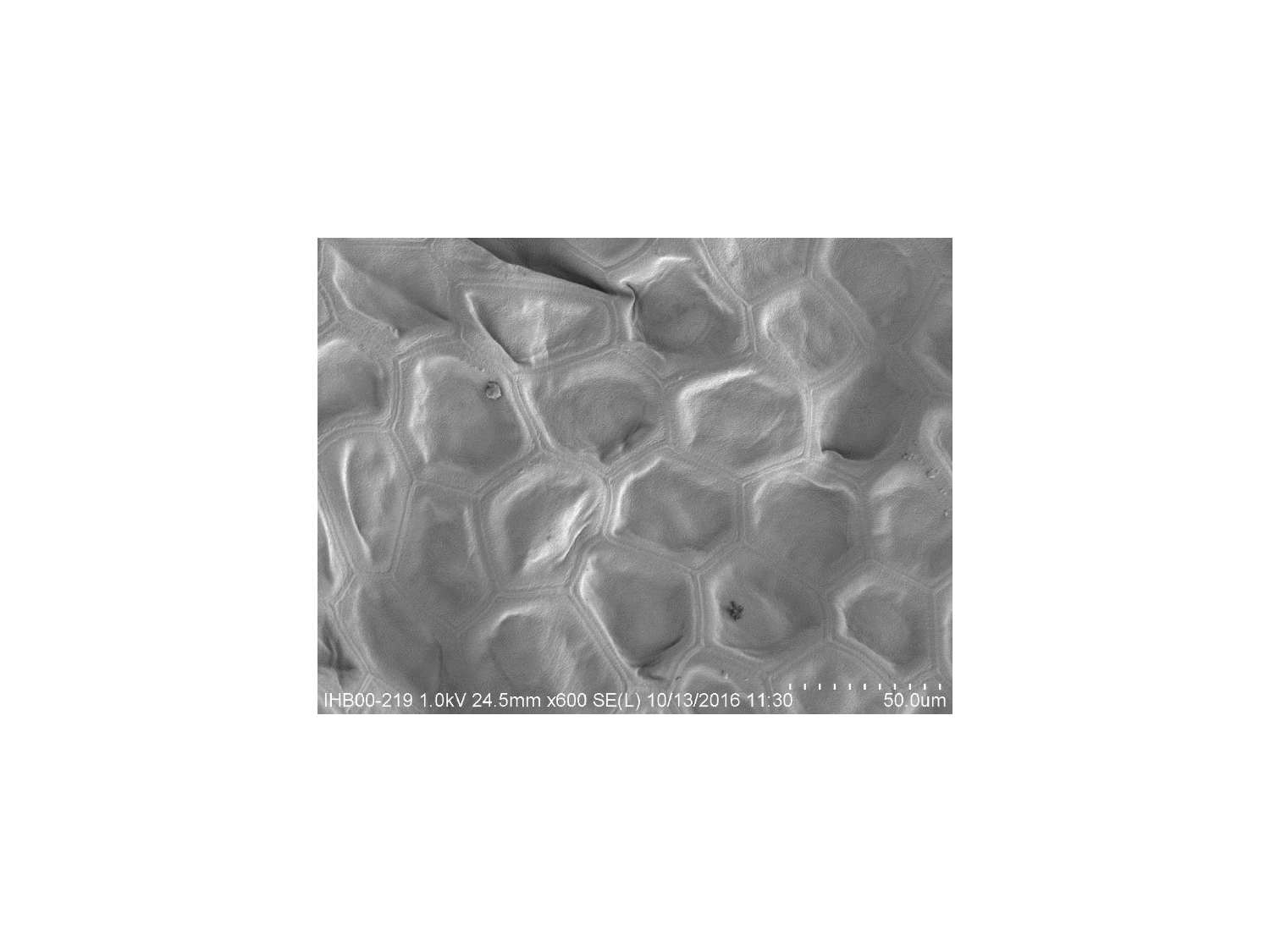

## Slide 5
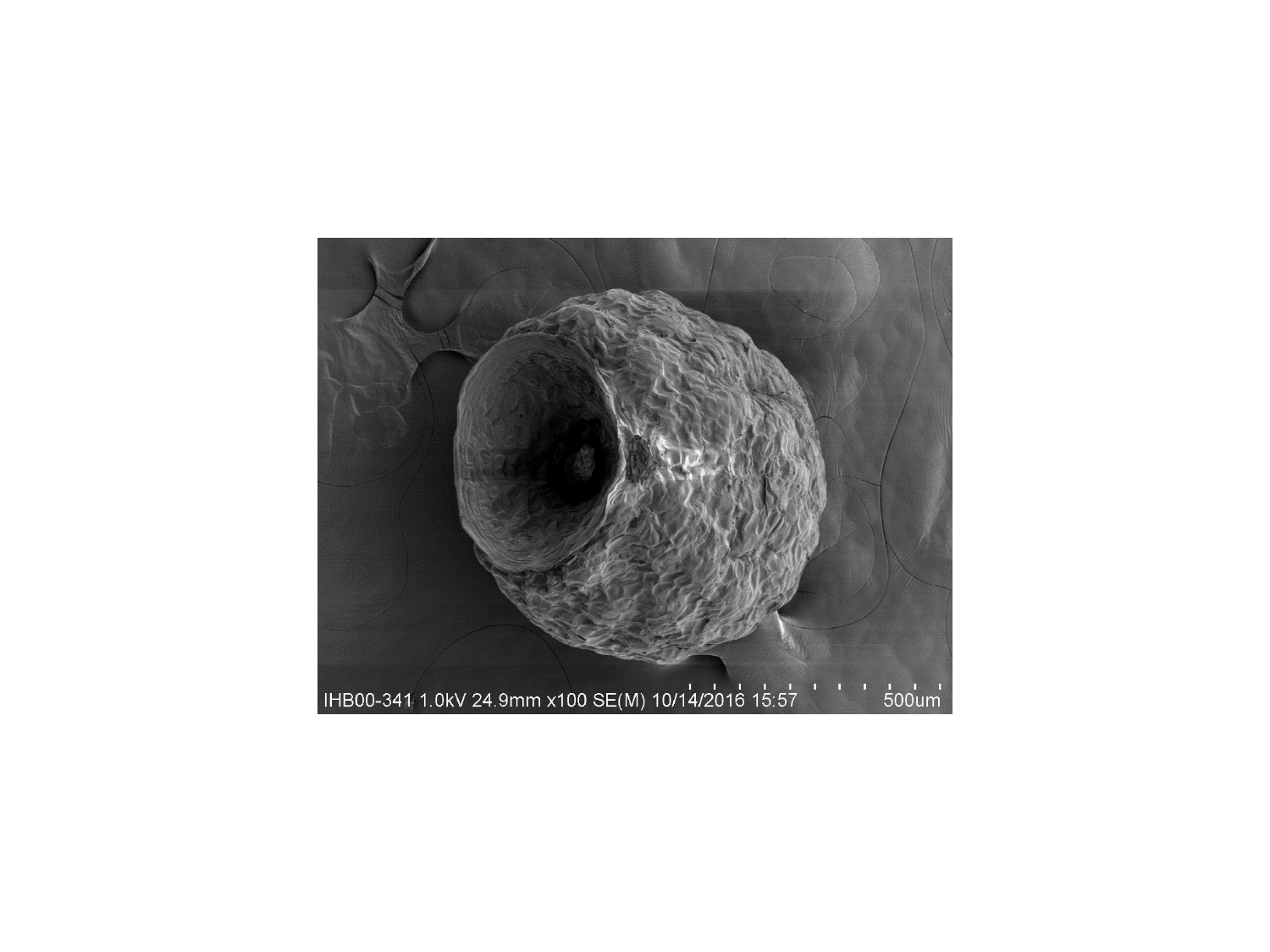

## Slide 6
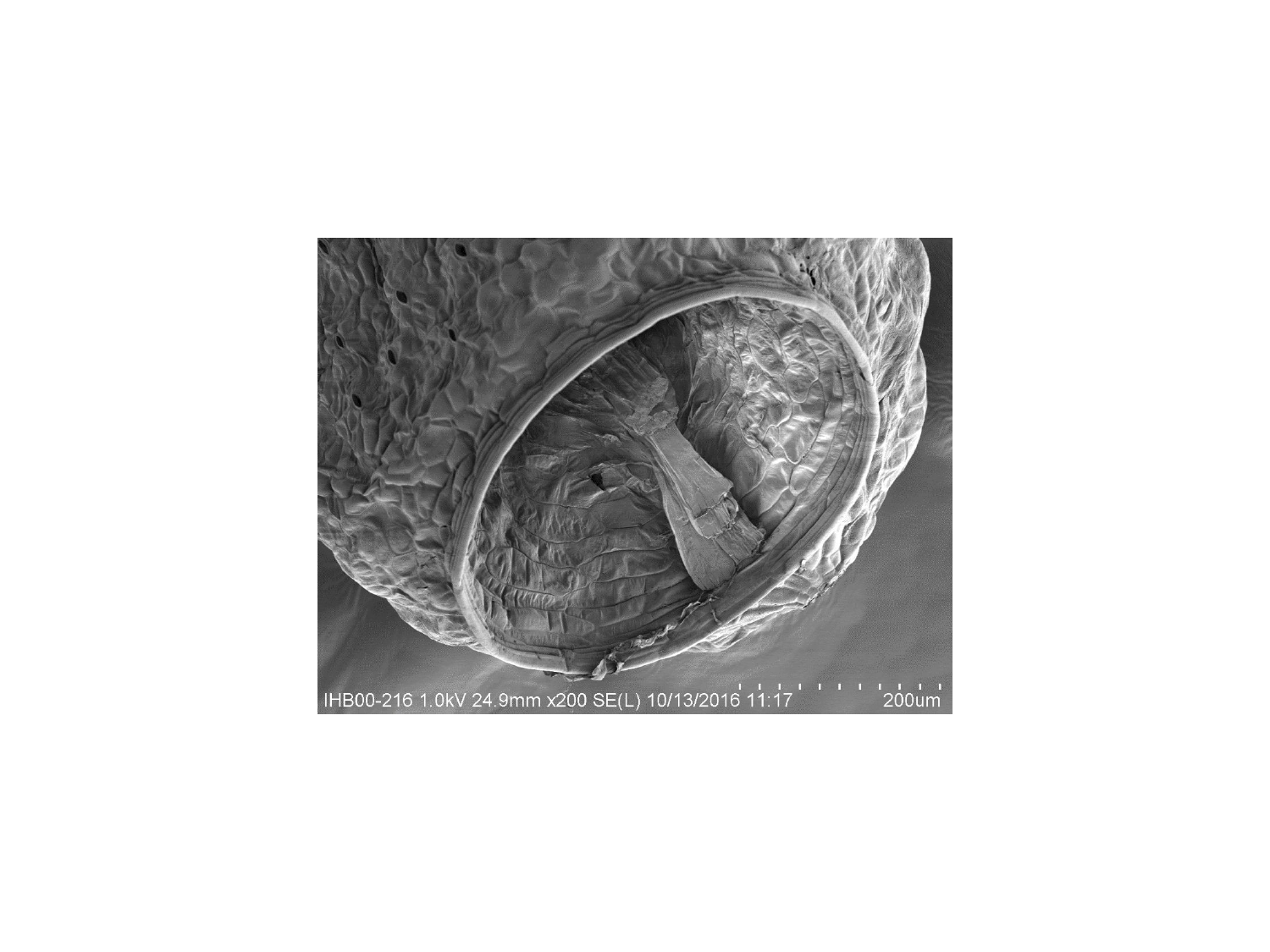

## Slide 7
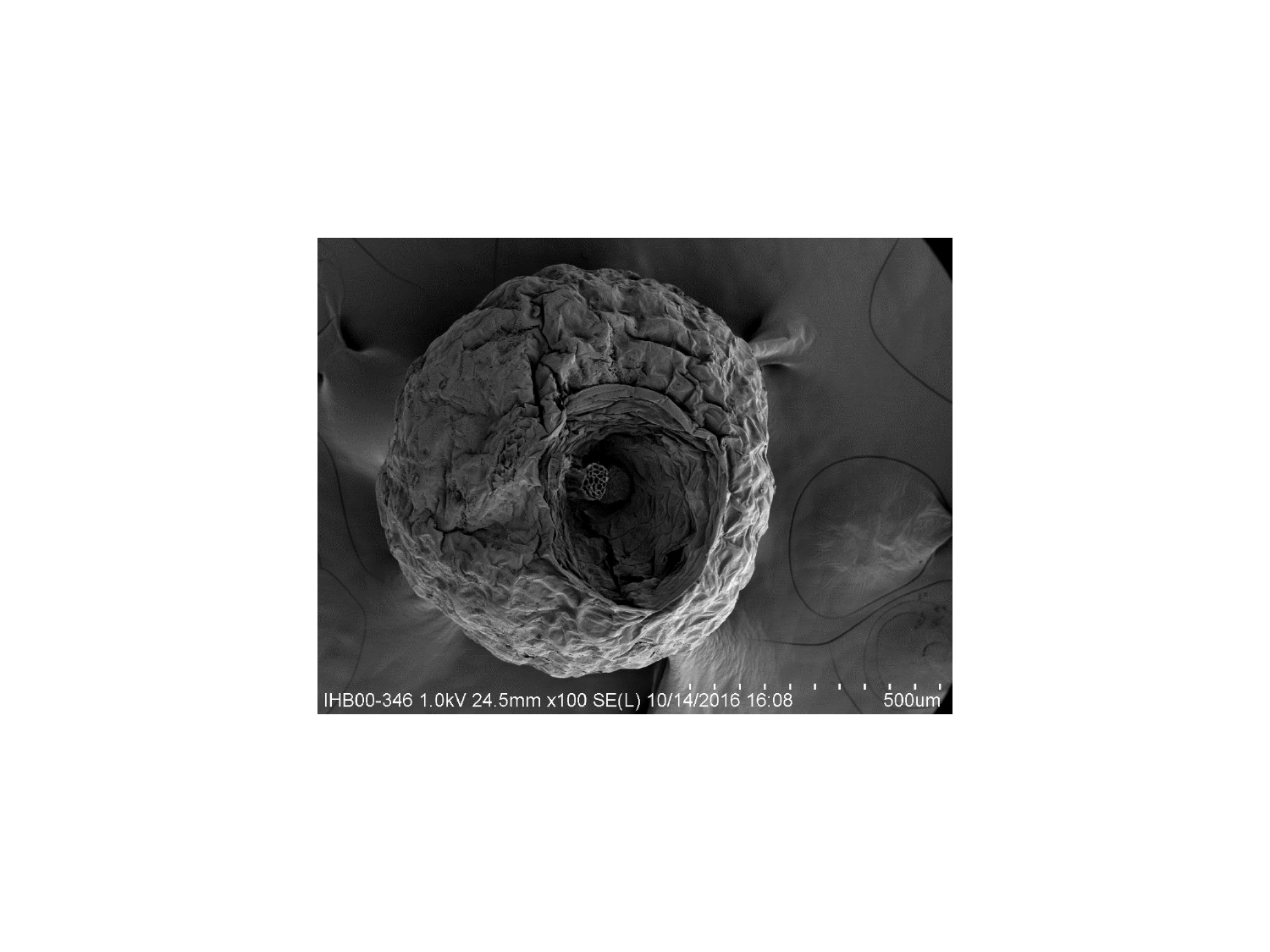

## Slide 8
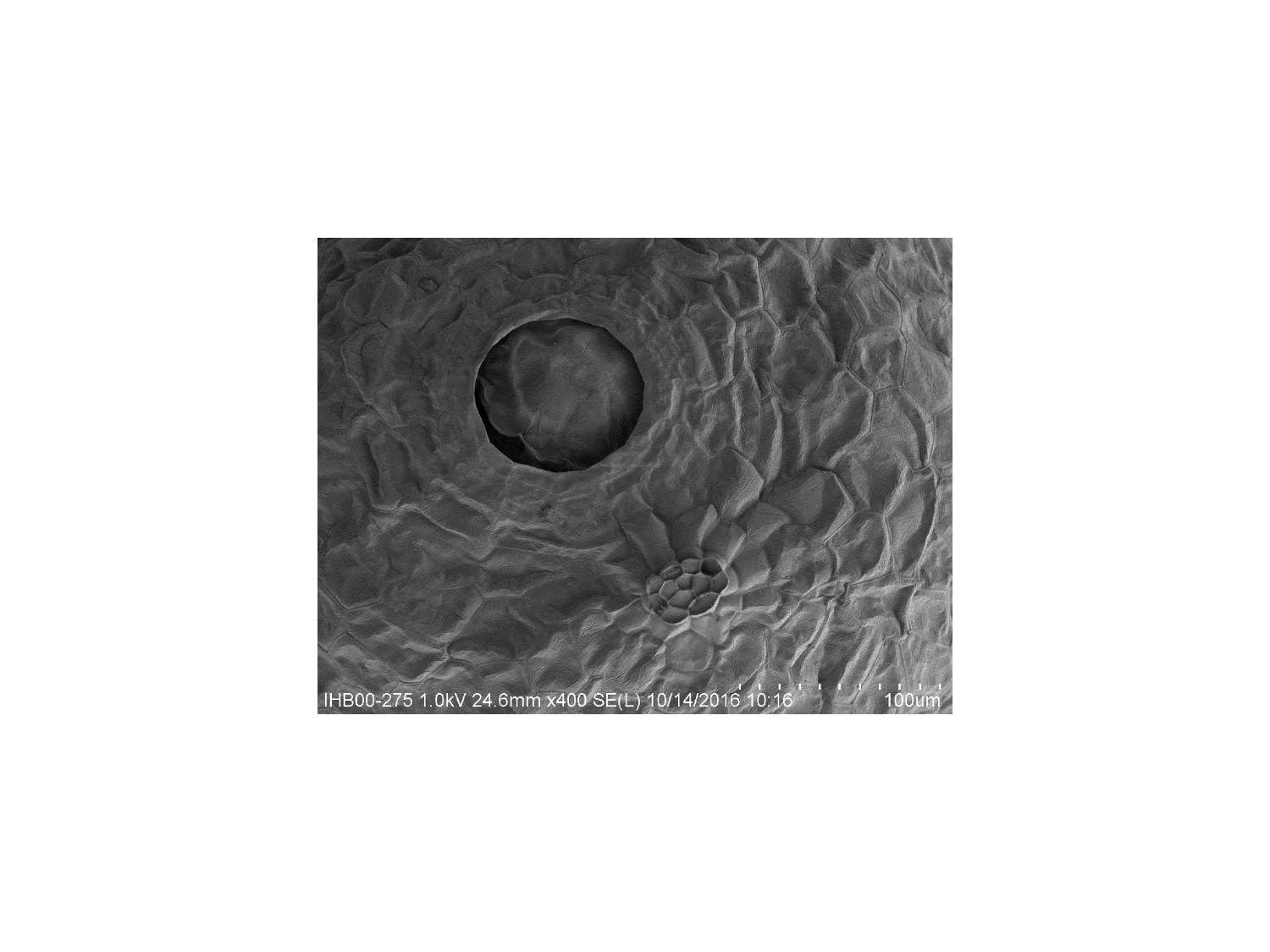

## Slide 9
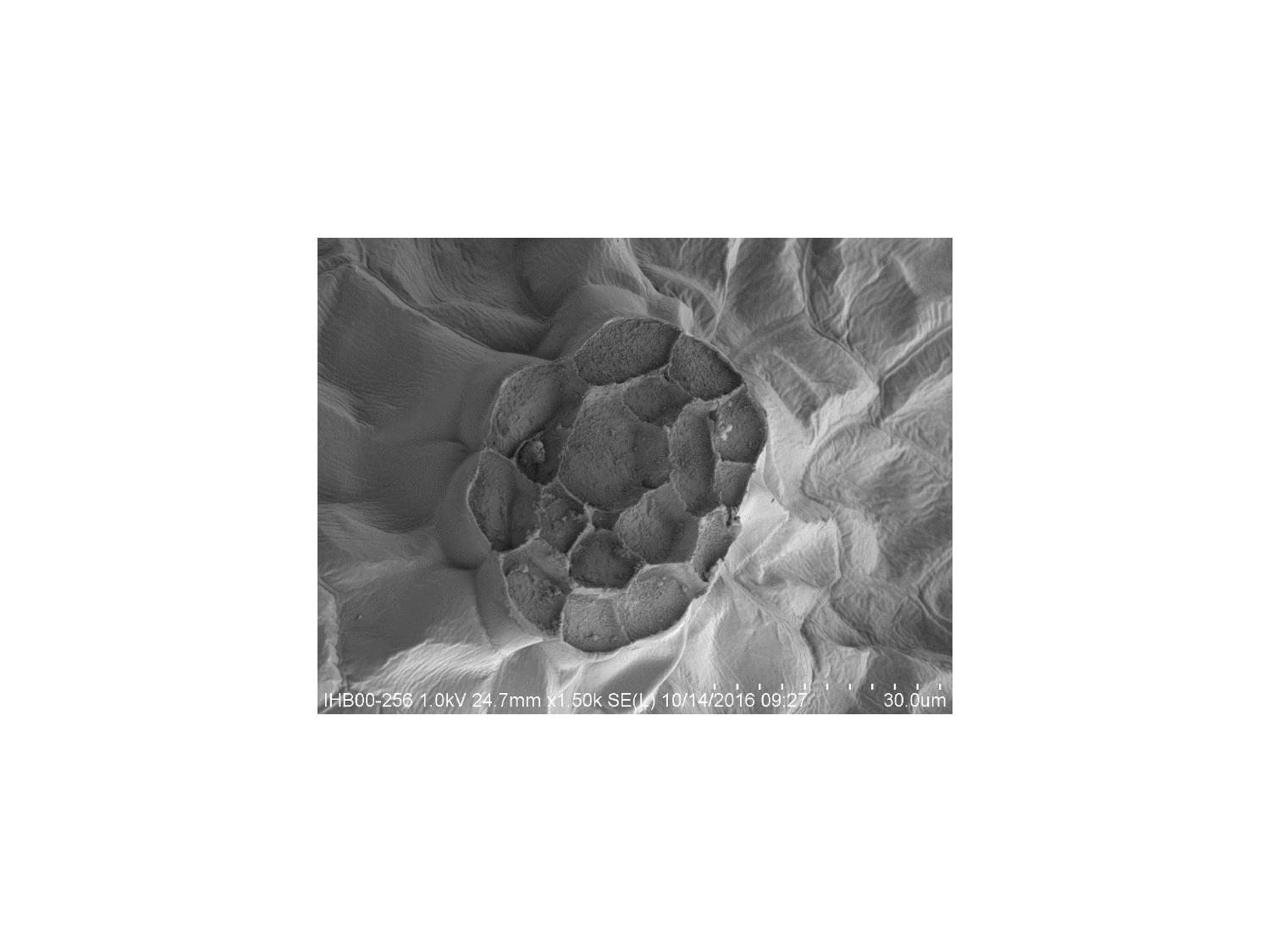

Supplement: Supplementary file 3 — Additional file 3: The raw data of Fig.2. [file 12870_2021_3165_MOESM3_ESM.pptx]

## Slide 1
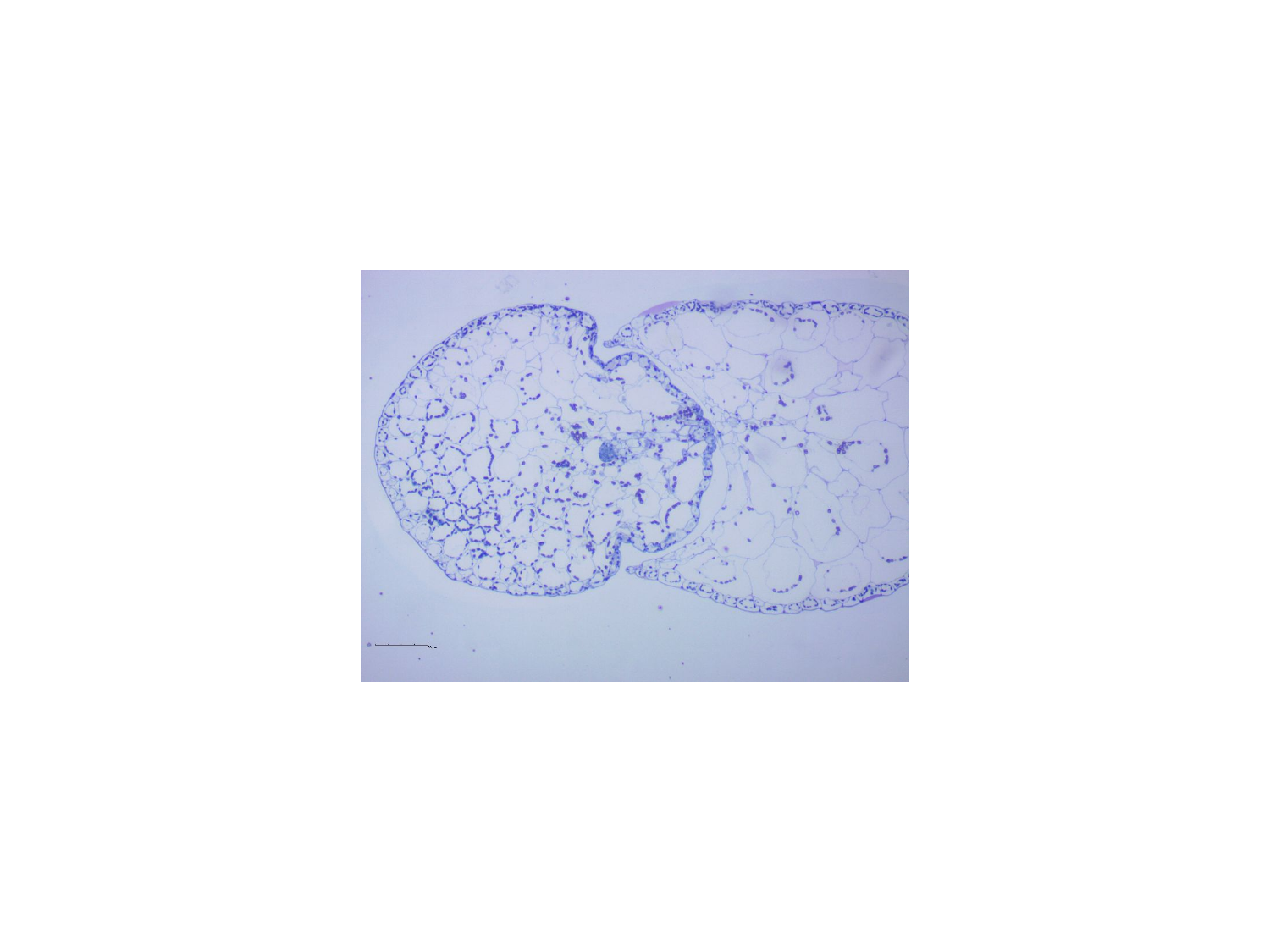

## Slide 2
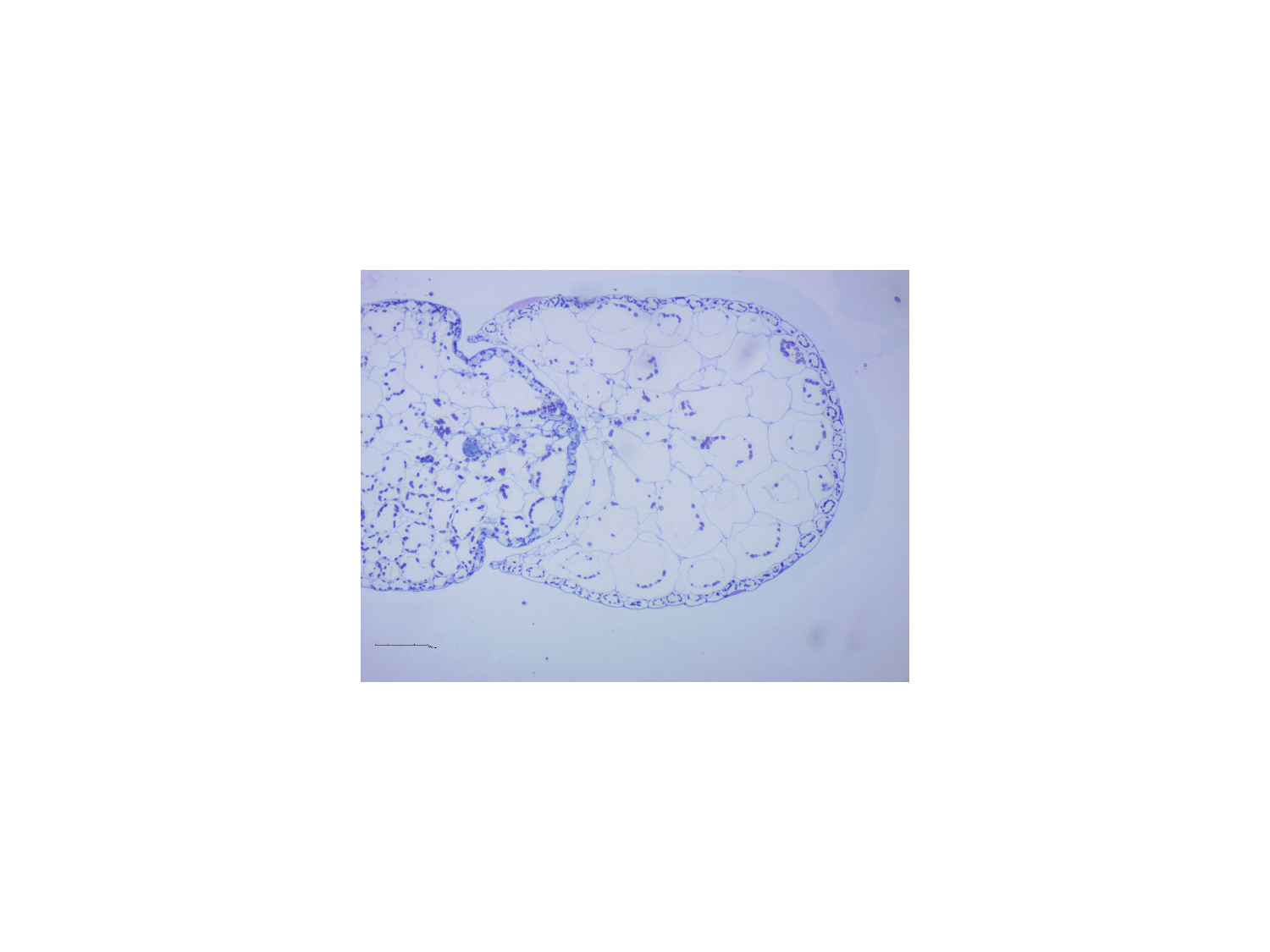

## Slide 3
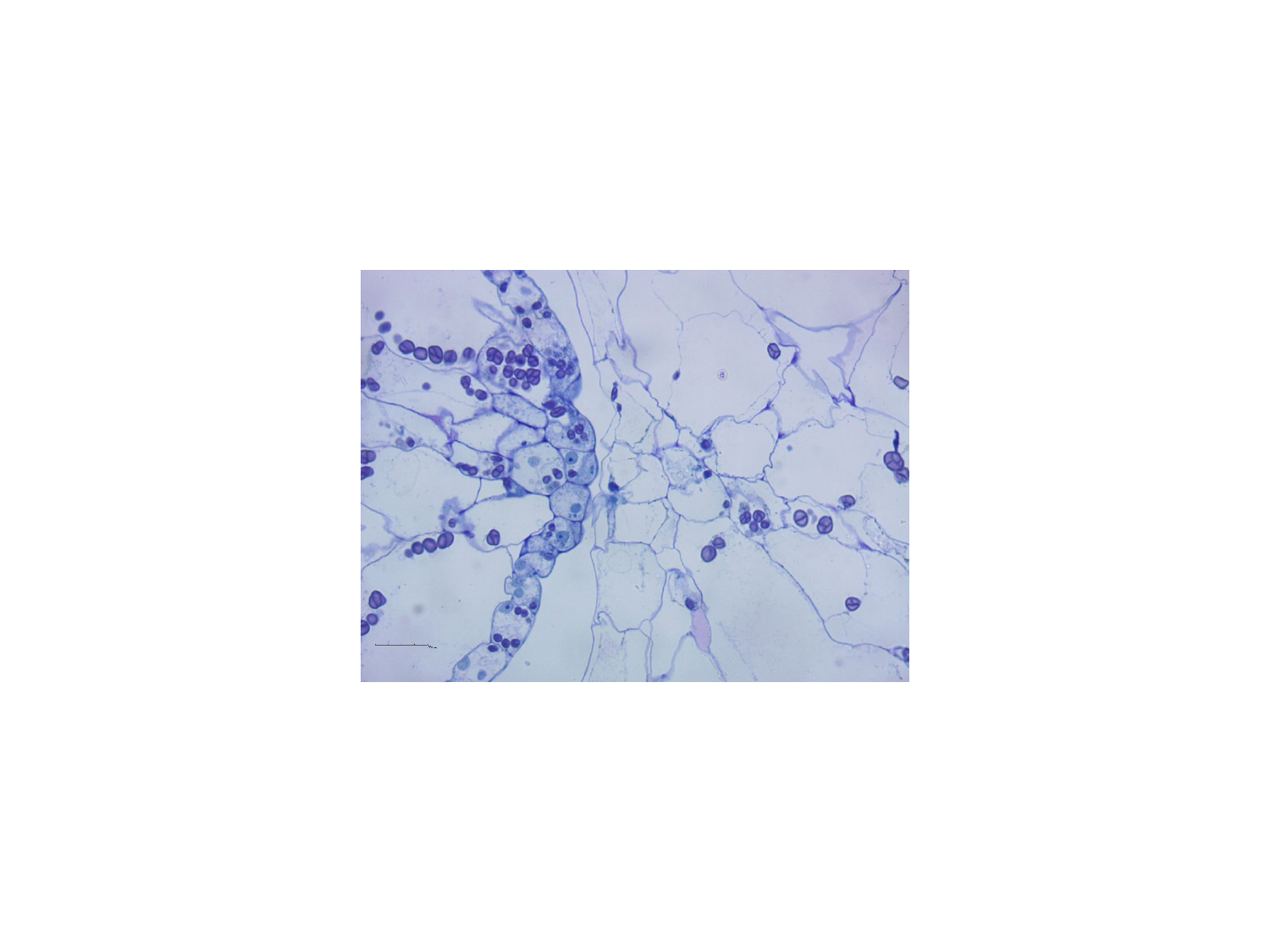

## Slide 4
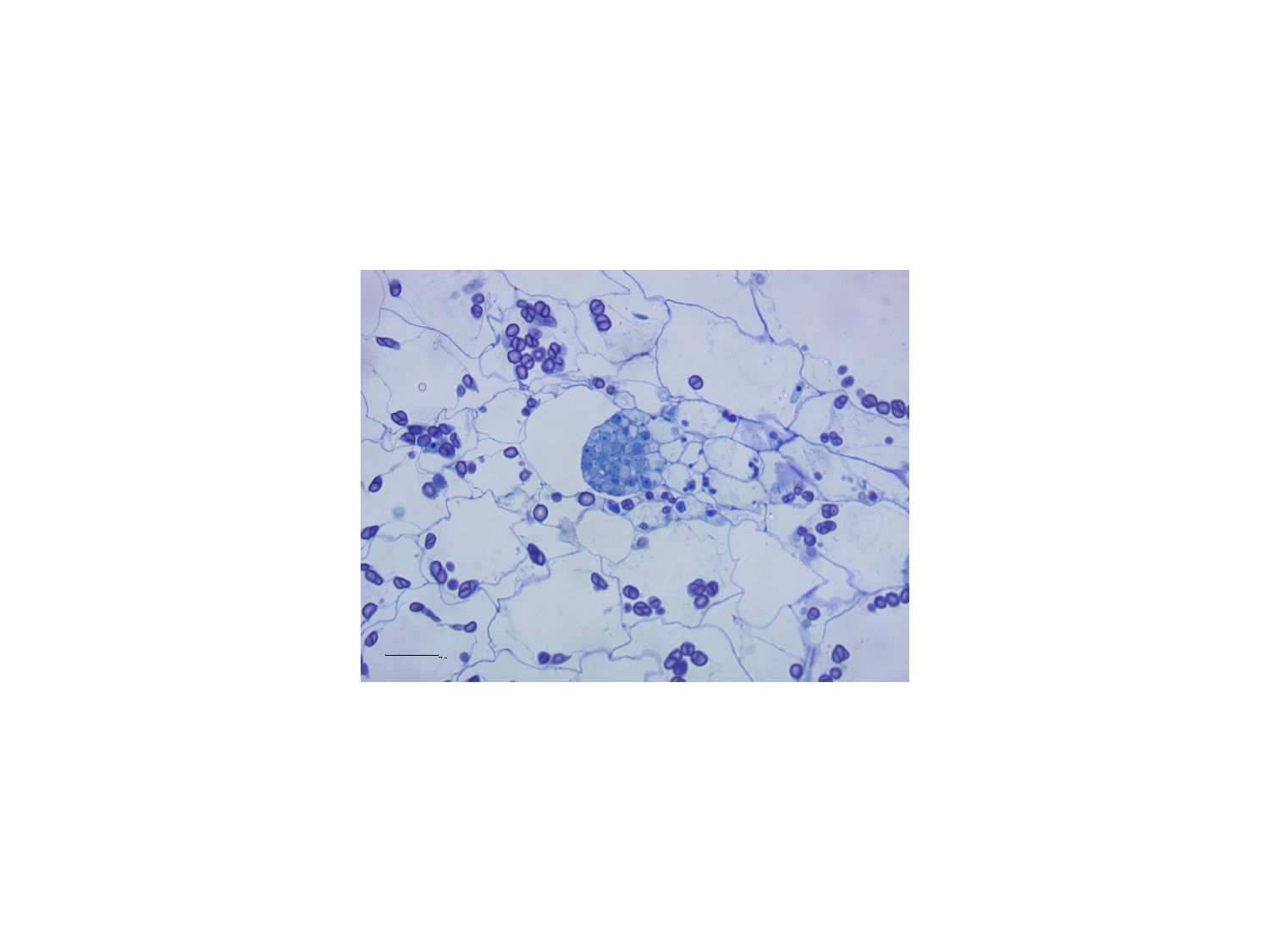

## Slide 5
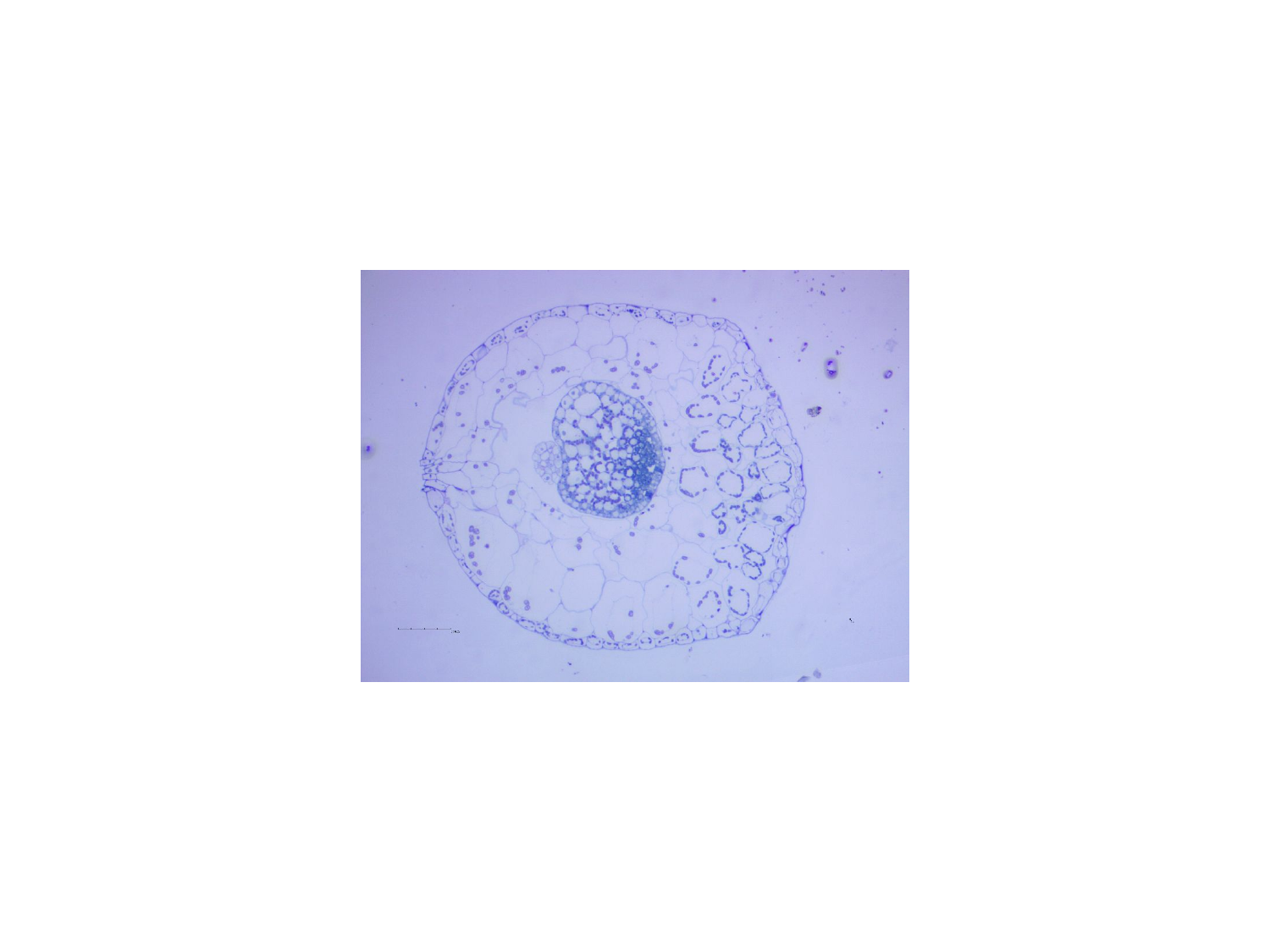

## Slide 6
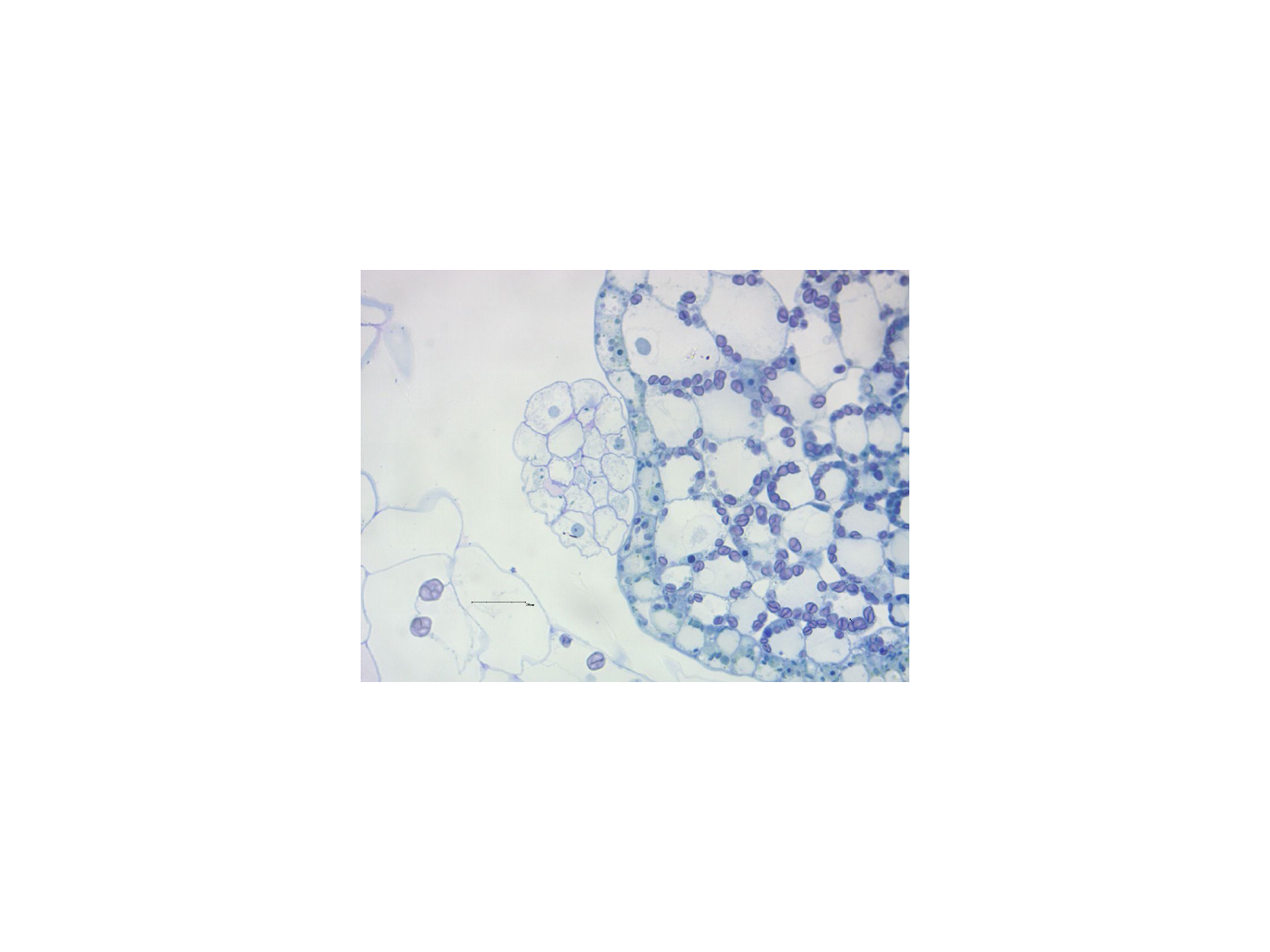

## Slide 7
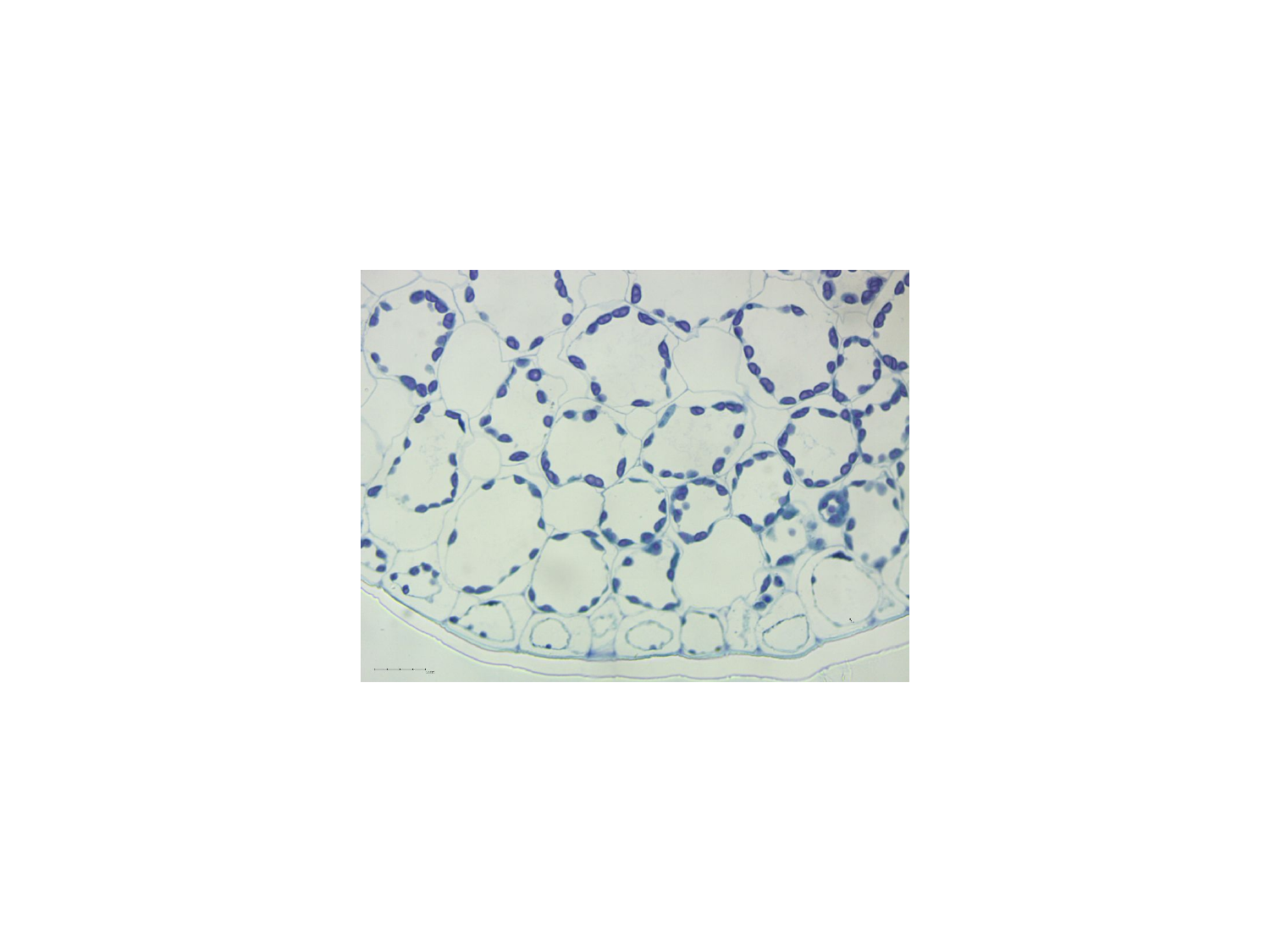

## Slide 8
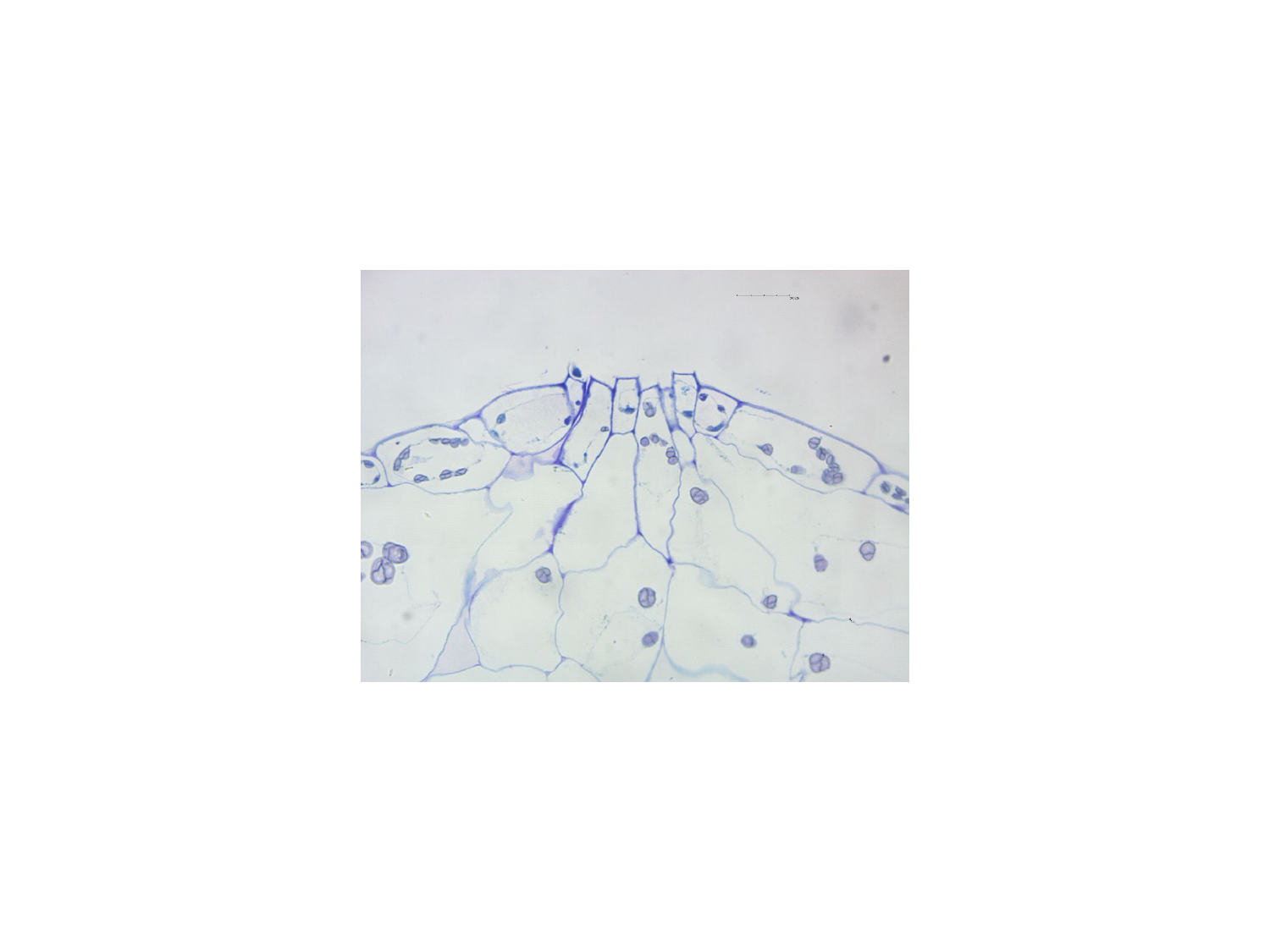

Supplement: Supplementary file 4 — Additional file 4: The raw data of Fig.3. [file 12870_2021_3165_MOESM4_ESM.pptx]

## Slide 1
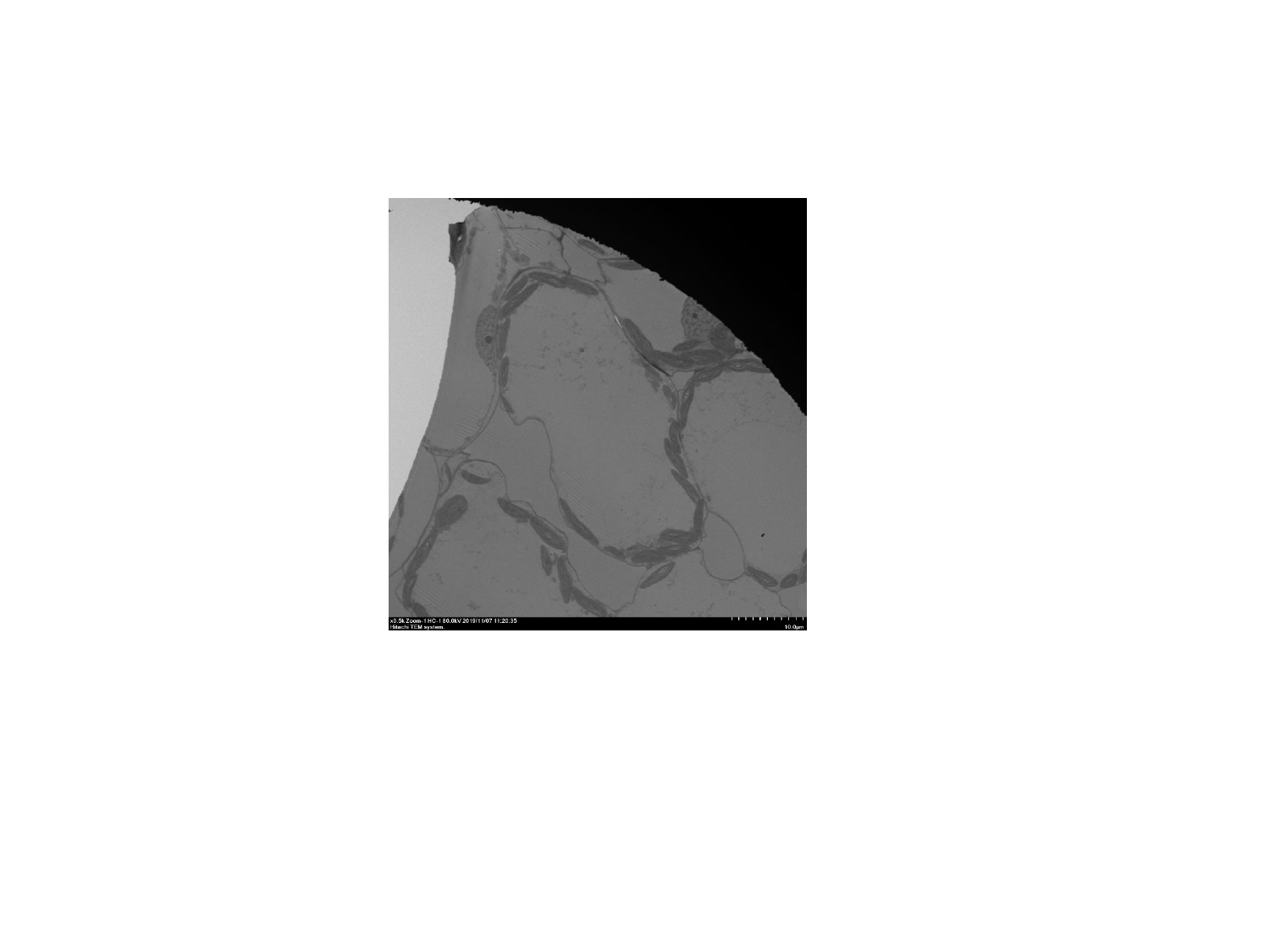

## Slide 2
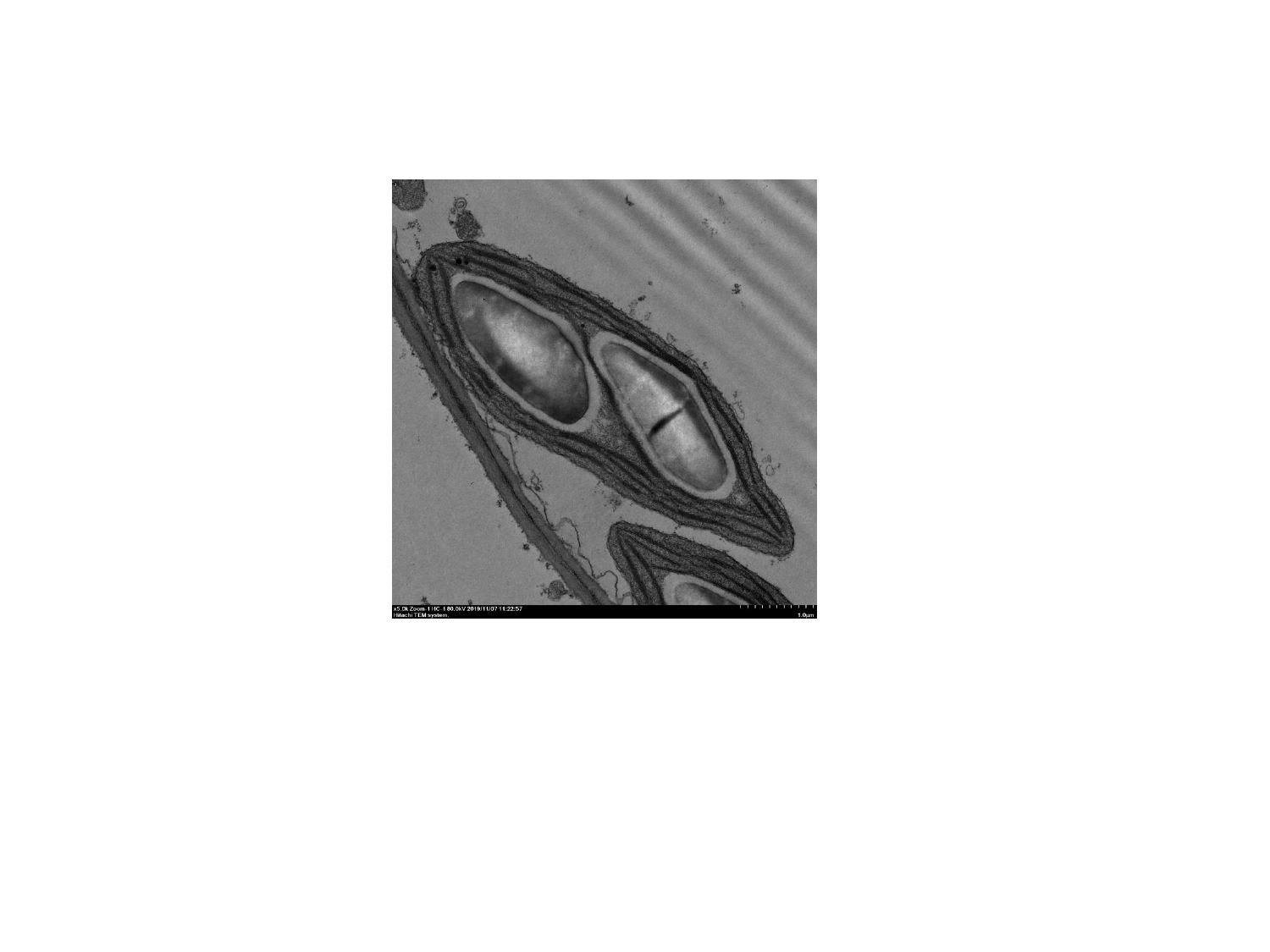

## Slide 3
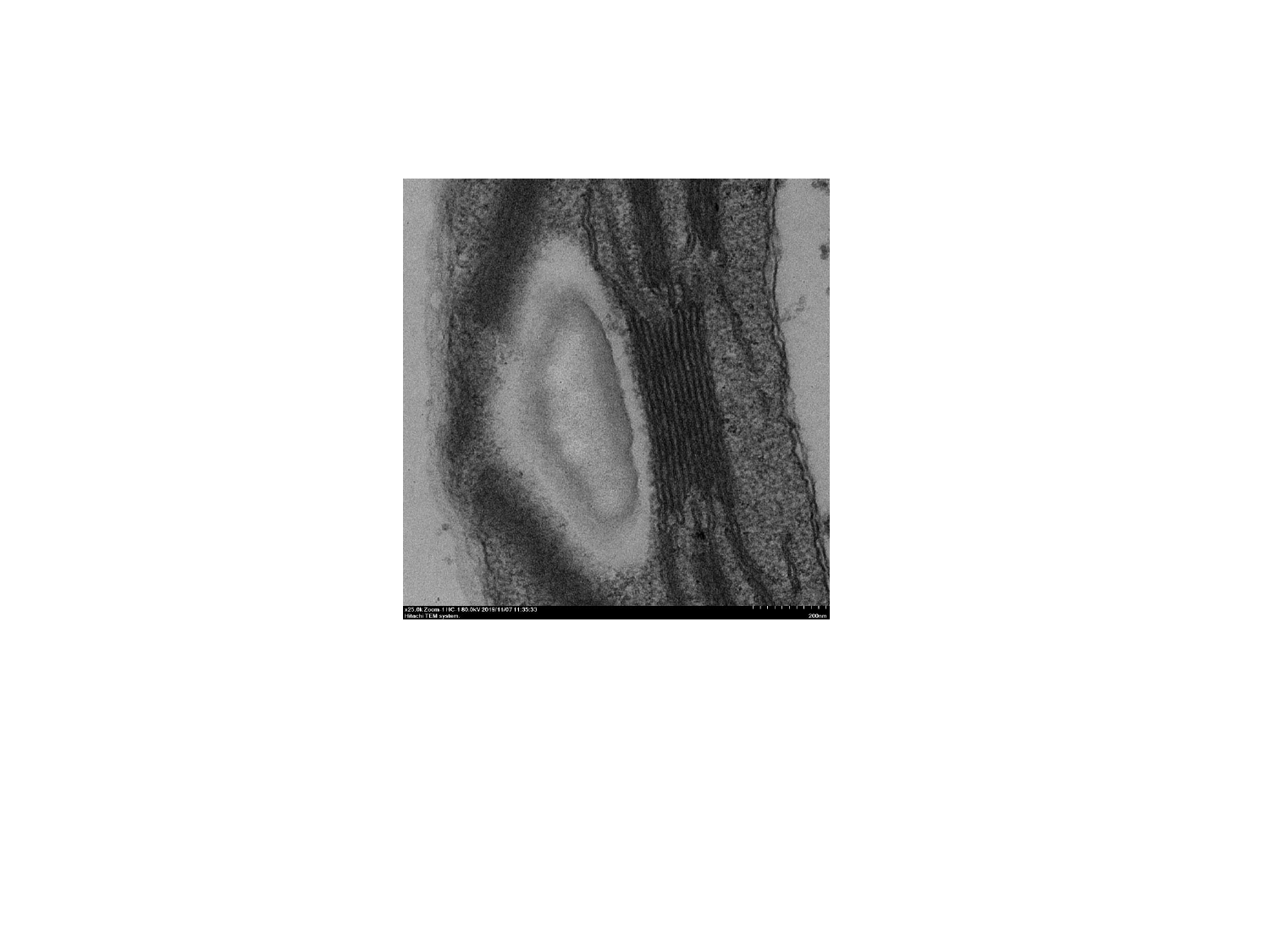

## Slide 4
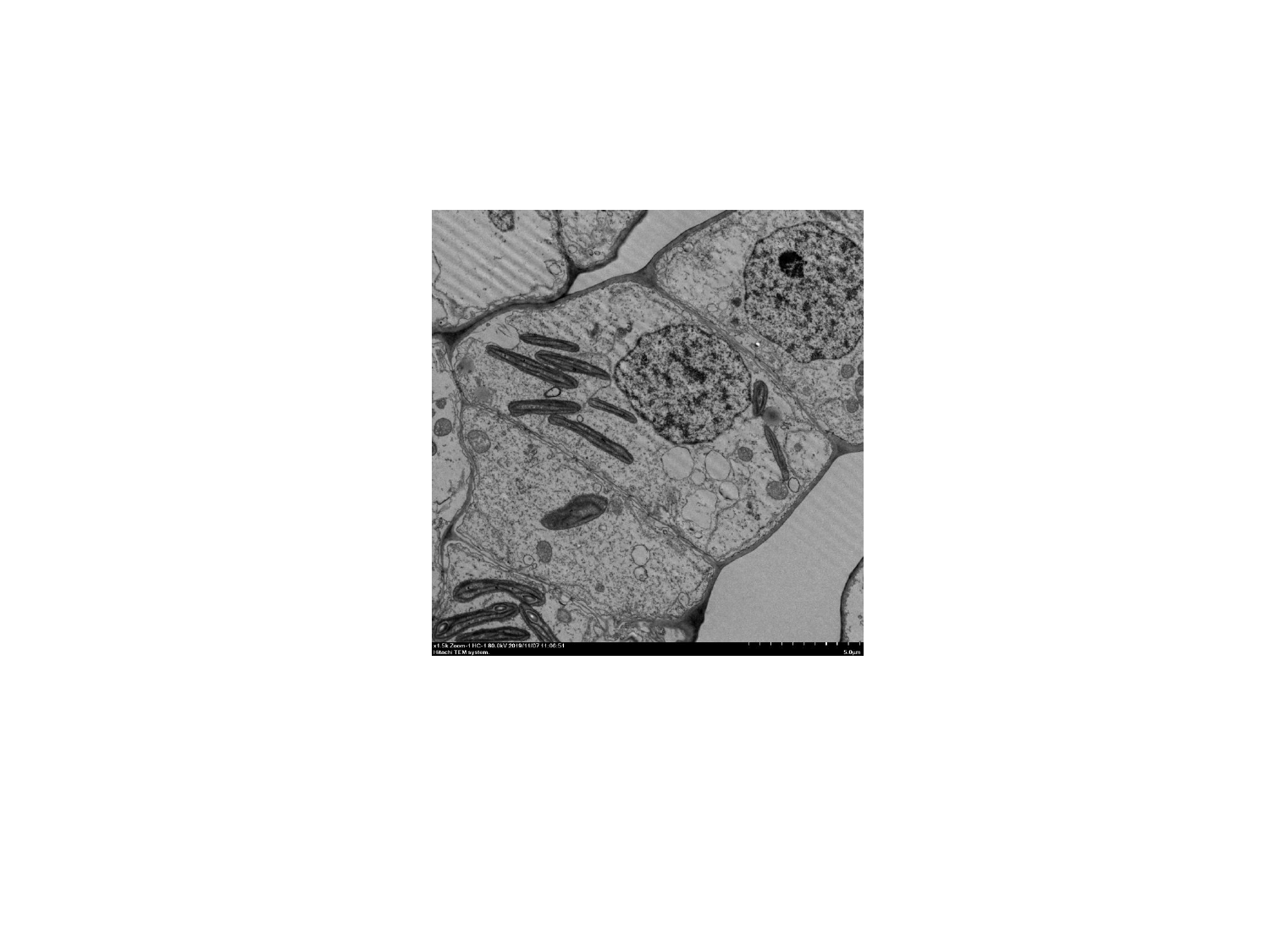

## Slide 5
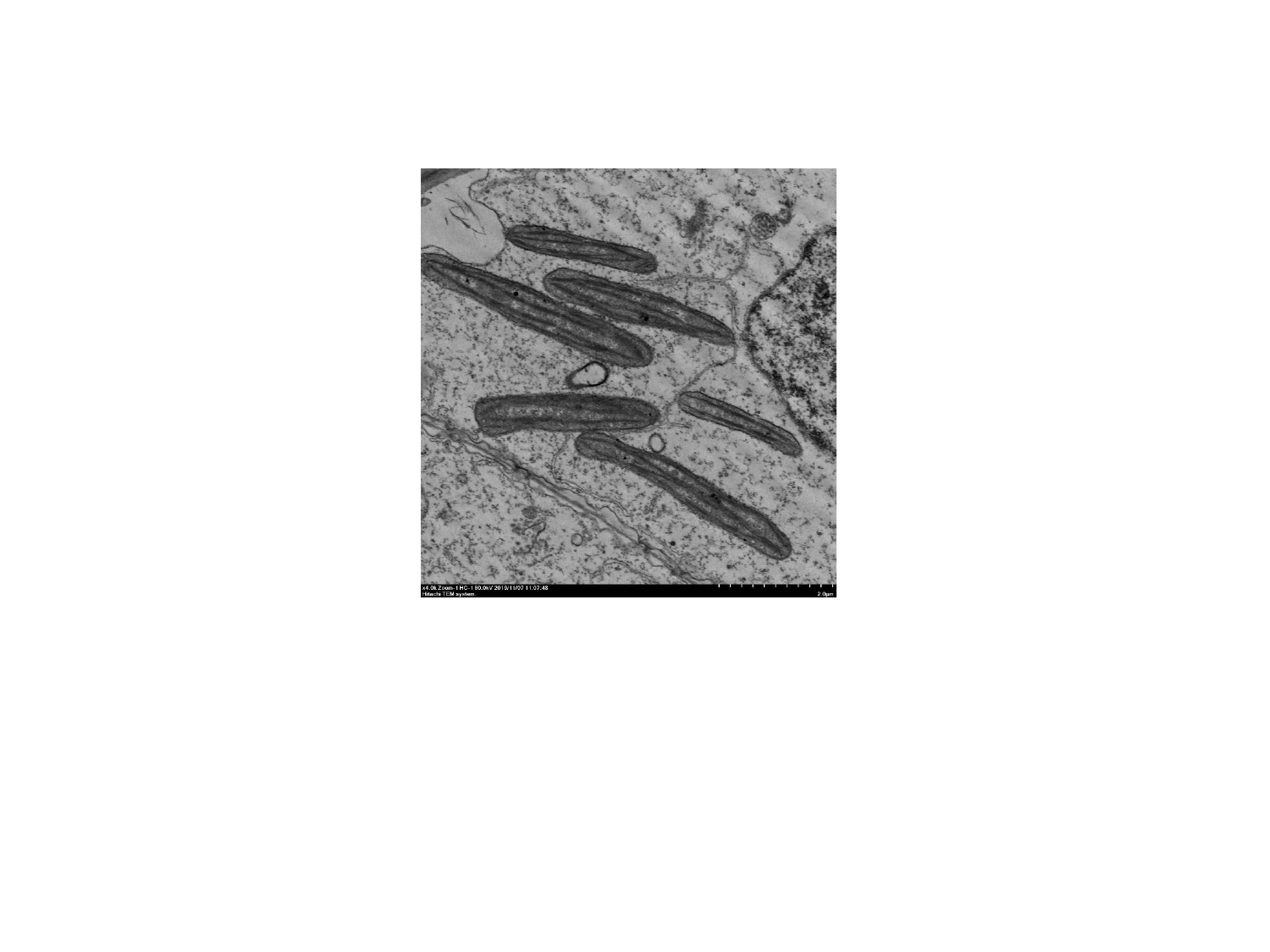

## Slide 6
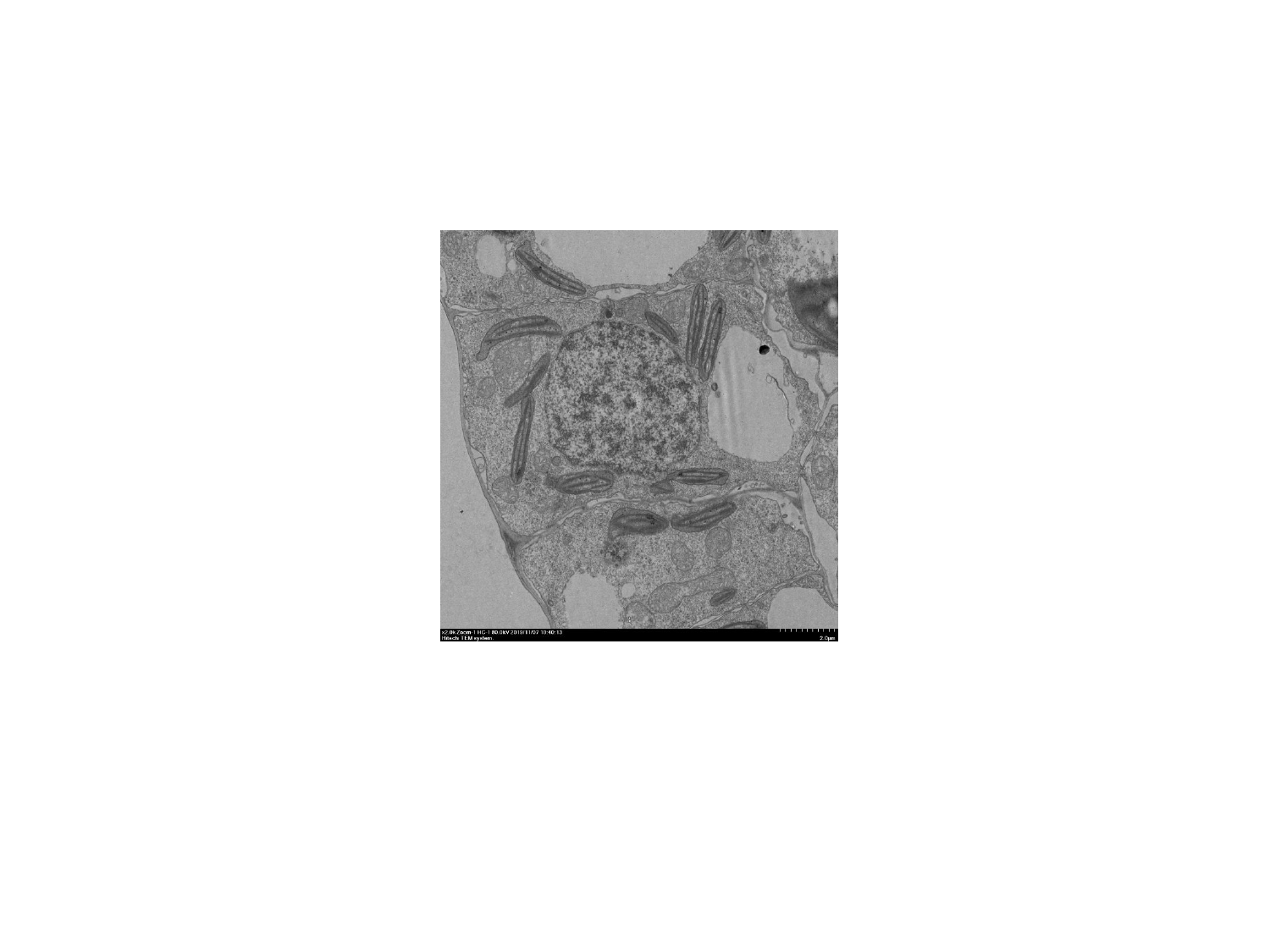

## Slide 7
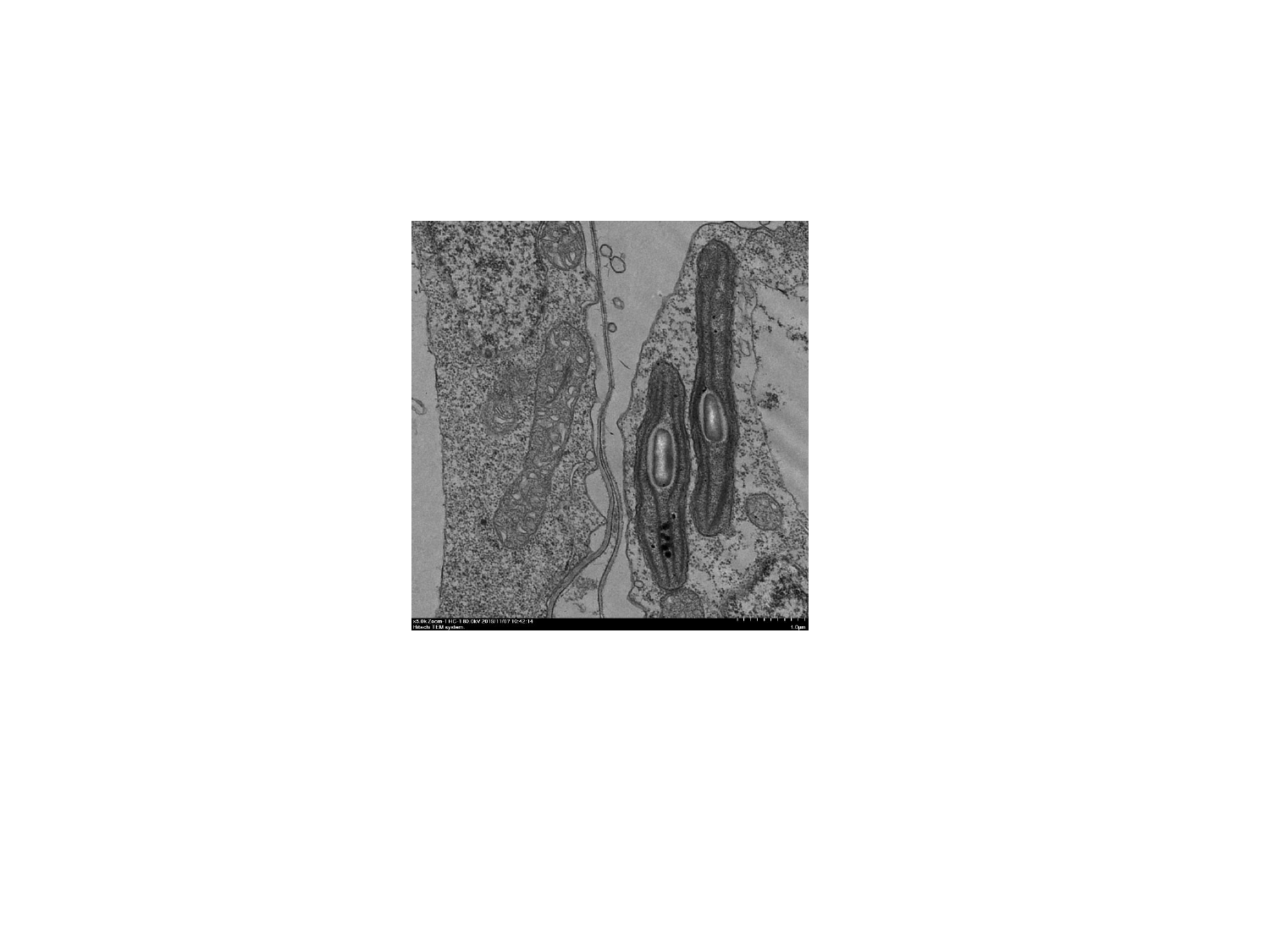

## Slide 8
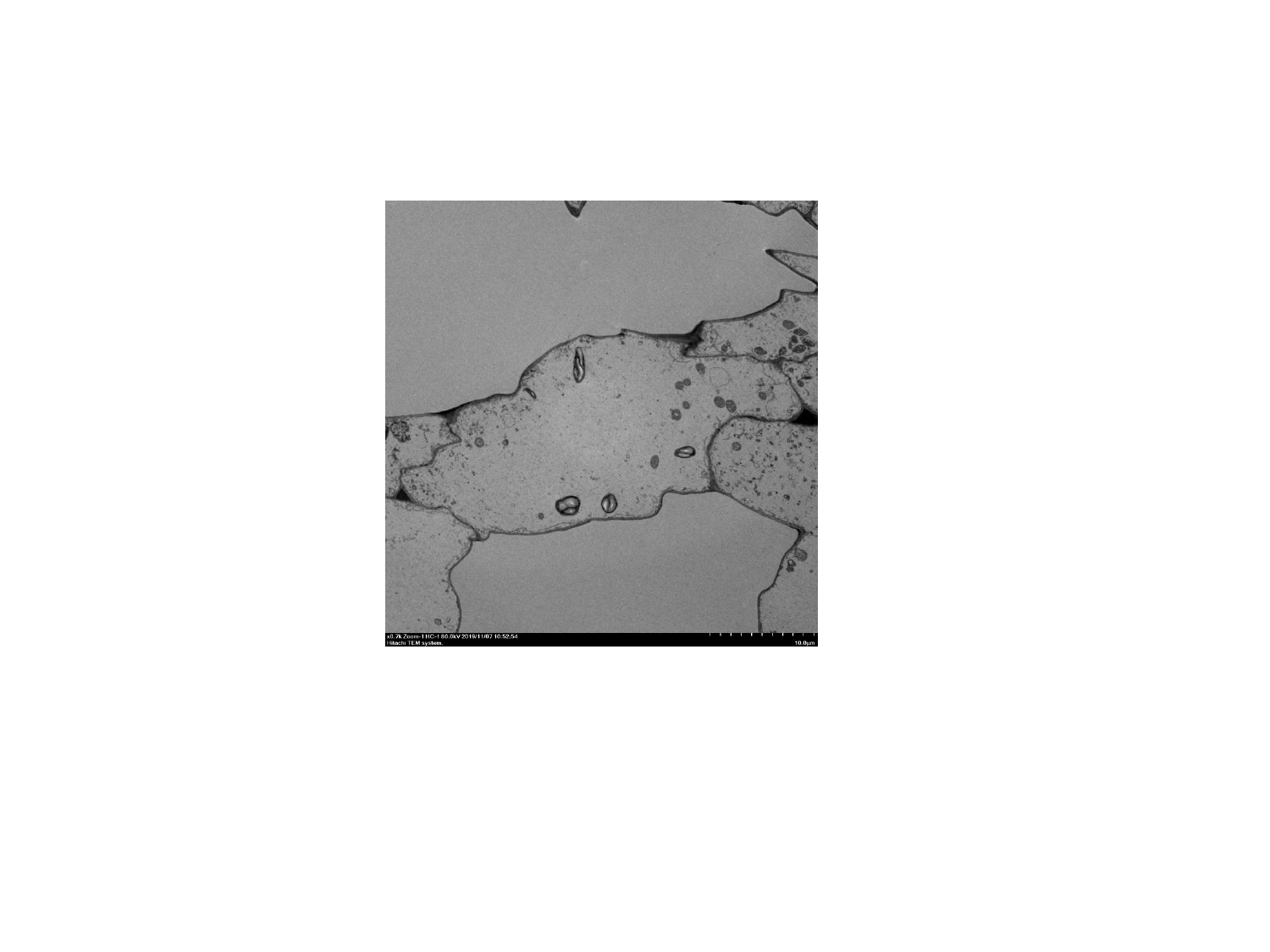

## Slide 9
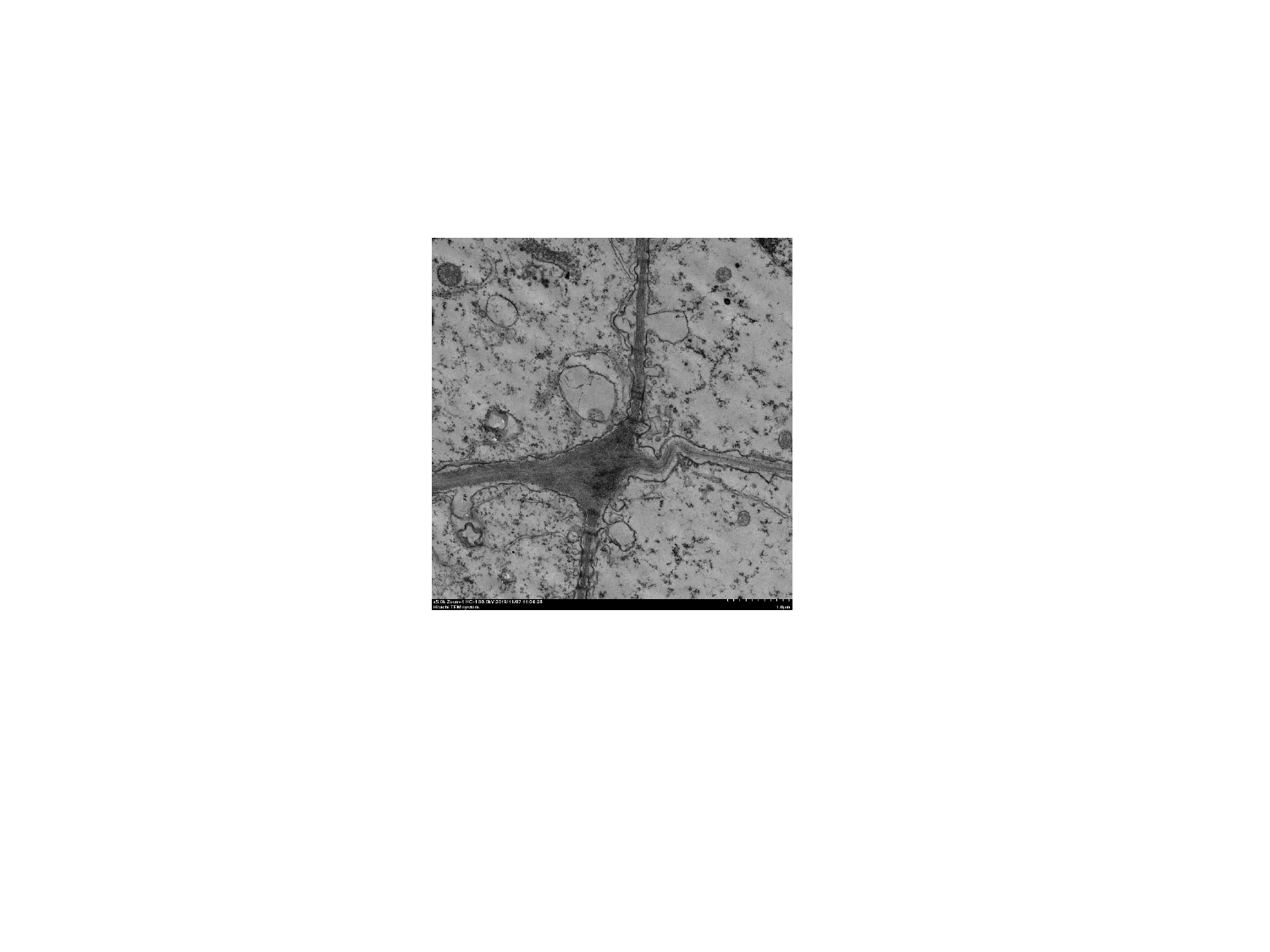

Supplement: Supplementary file 5 — Additional file 5: The raw data of Fig.4. [file 12870_2021_3165_MOESM5_ESM.pptx]
